# Indole-3-Carbaldehyde from *Limosilactobacillus reuteri* Boosts Chemotherapy Response in Diffuse Large B Cell Lymphoma by Blocking the Mechanistic Target of Rapamycin Pathway

**DOI:** 10.34133/research.1267

**Published:** 2026-05-04

**Authors:** Zhengfeng Zhang, Congcong Li, Yuhan Tang, Bingrong Liu, Jingyu Wang, Leilei Kong, Wanwan Bao, Hurong Lai, Tingtao Chen, Jian Li

**Affiliations:** ^1^ Department of Hematology, The Second Affiliated Hospital, Jiangxi Medical College, Nanchang University, Nanchang, Jiangxi, China.; ^2^ Jiangxi Province Key Laboratory of Bioengineering Drugs, School of Pharmacy, Jiangxi Medical College, Nanchang University, Nanchang, Jiangxi, China.; ^3^ National Engineering Research Center for Bioengineering Drugs and the Technologies, Institute of Translational Medicine, Jiangxi Medical College, Nanchang University, Nanchang, Jiangxi, China.; ^4^ Department of Cardiovascular Surgery, The Second Affiliated Hospital, Jiangxi Medical College, Nanchang University, Nanchang, Jiangxi, China.; ^5^ Department of Medical Oncology, Harbin Medical University Cancer Hospital, Harbin, China.; ^6^ Jiangxi Sanxin Medtec Co. Ltd., Nanchang, Jiangxi 330200, China.; ^7^ Department of Clinical Trial Research Center, The Second Affiliated Hospital of Nanchang University, Nanchang, Jiangxi, China.; ^8^ Jiangxi Provincial Key Laboratory of Hematological Diseases, The Second Affiliated Hospital, Jiangxi Medical College, Nanchang University, Nanchang, Jiangxi, China.

## Abstract

Diffuse large B cell lymphoma (DLBCL) presents a critical clinical challenge due to declining chemosensitivity and difficult-to-manage dose-limiting toxicities. Although gut microbiota modulation shows potential for “toxicity reduction and efficacy enhancement”, its mechanism in DLBCL remains unclear. Comparative analysis revealed a marked reduction of beneficial bacteria in patients with DLBCL versus healthy volunteers, with a marked decrease in the abundance of core probiotics, particularly *Limosilactobacillus reuteri*. Fecal microbiota transplantation from healthy donors into DLBCL mouse models reduced tumor burden, improved chemosensitivity, and alleviated intestinal toxicity. A core probiotic strain, *L. reuteri* HG001, was isolated and shown to replicate these effects alone, with the tryptophan metabolite indole-3-carbaldehyde (ICAld) identified as the key component responsible for its adjunctive antitumor activity. Mechanistic studies demonstrated that ICAld exerts significant adjunctive antitumor effects both *in vitro* and *in vivo* in a dose-dependent manner in mouse models; it acts by activating the aryl hydrocarbon receptor (AHR)/cytochrome P450 family 1 subfamily A member 1 (CYP1A1)/reactive oxygen species (ROS) axis, inhibiting the phosphatidylinositol 3-kinase (PI3K)/AKT/mechanistic target of rapamycin (mTOR) signaling pathway, promoting apoptosis, and synergizing with cyclophosphamide. An aryl hydrocarbon receptor antagonist reversed both the chemosensitizing and intestinal protective effects of *L. reuteri* HG001 and ICAld. This study elucidates a microbiota-mediated mechanism in DLBCL and supports *L. reuteri* HG001 as a probiotic adjuvant to enhance therapy while reducing toxicity.

## Introduction

Diffuse large B cell lymphoma (DLBCL) is a highly aggressive non-Hodgkin lymphoma characterized by the malignant clonal proliferation of B lymphocytes. The disease has an insidious onset and rapid progression, with untreated patients typically having a median survival of less than 12 months [1,2]. Currently, the R-CHOP regimen (rituximab combined with cyclophosphamide (CTX), doxorubicin, vincristine, and prednisone) is the standard first-line treatment strategy, benefiting approximately 60% of patients; however, due to treatment-related toxicities and tumor molecular heterogeneity, about 40% of patients eventually experience relapse or treatment resistance [3,4]. Consequently, the pursuit of novel treatment targets and intervention methods to further improve the prognosis of patients with DLBCL is now a key area of ongoing clinical investigation.

The intestinal microbiome plays a vital role in regulating and maintaining the host’s immune response, with imbalances or irregularities in its function closely linked to tumor development and treatment outcomes [2,5–8]. Gopalakrishnan et al. [9] examined variations in the gut microbiome of patients with melanoma undergoing programmed cell death protein 1 inhibitor therapy, revealing that patients who responded positively to programmed cell death protein 1 inhibitors had higher levels of *Clostridia* and *Ruminococcaceae.* At the same time, *Bacteroides* made up a smaller proportion of their gut microbiome. Conversely, individuals with weaker responses showed the opposite pattern; fecal microbiota transplantation (FMT) from those with strong responses into weaker responders significantly mitigates their adverse reactions; this effect is notably due to increased activation of CD8^+^ T cells linked to anticancer immunity, elevated levels of immune checkpoint molecules such as T cell immunoreceptor with immunoglobulin and immunoreceptor tyrosine-based inhibition motif domains (TIGIT), T-box expressed in T cells (T-bet), and lymphocyte activation gene 3 (LAG-3), and a decrease in tumor-associated myeloid cells [10–12]. This suggests a strong connection between gut microbiome composition and the success of cancer treatments. Importantly, differences in the intestinal microbiome are also reflected in blood-related cancers. Yoon et al. [2] and Li et al. [13] analyzed gut microbiota differences between patients with DLBCL and healthy individuals, finding a significant decrease in α-diversity of these microbiota; they also observed an increase in *Enterobacteriaceae* level, which was closely associated with reduced survival without disease progression, higher recurrence rates, and more severe treatment-related toxic effects. These findings suggest that variations in gut microbiota have significant clinical implications for the diagnosis and treatment of blood cancers. Although current studies have firmly established the potential influence of gut microbiota variation on various cancers, data on managing and controlling gut microbiota in DLBCL therapy, along with its precise mechanisms, remain quite limited.

Probiotics, a microbial formulation that can regulate gut equilibrium, enhance immune responses, and modify metabolic processes, exhibit significant potential in complementary therapies for diseases such as gynecological diseases, diabetes, obesity, and cancer [14]. *Limosilactobacillus reuteri* has garnered considerable attention as a focus of research in microbe-based adjunctive therapy due to its ability to produce various metabolites, such as reuterin and indole derivatives, which hold substantial promise for improving the prognosis of diseases including ulcerative colitis, atherosclerosis, and *Helicobacter pylori* infection [15,16]. Furthermore, microencapsulation with whey protein and gum arabic effectively enhances the viability of this strain under gastrointestinal conditions, thermal stress, and refrigerated storage, consistently maintaining viable counts above the recommended threshold required for probiotic efficacy [17]. The study by Bender et al. [18] showed that *L. reuteri* boosts the effectiveness of immune checkpoint inhibitors in a type 1 cytotoxic T cell-dependent way by emitting indole-3-carbaldehyde (ICAld), which activates the aryl hydrocarbon receptor (AHR) in CD8^+^ T cells. In addition, reuterin influences the reactive oxygen species (ROS)–hypoxia-inducible factor-1α (HIF-1α) signaling pathway by increasing reuterin production, boosting glycerophospholipid metabolism, elevating arachidonic acid levels, and triggering the trained immune response in tumor-associated macrophages, thereby further hindering melanoma progression [19]. Moreover, reuterin produced by a strain of *L. reuteri* isolated from infant feces has been confirmed to exhibit broad-spectrum antibacterial activity against a variety of Gram-positive and Gram-negative pathogens as well as foodborne spoilage bacteria [20]. Current research has conclusively demonstrated that *L. reuteri*, through its combined “microbiota-metabolism-immune” network, can significantly enhance the efficacy of cancer chemotherapy and immunotherapy. Given the previously mentioned process, *L. reuteri* shows promise as a treatment option, potentially enhancing the effectiveness of DLBCL chemotherapy and improving the disease outlook.

This research involved an extensive examination of gut microbiota and metabolites in both patients with DLBCL and healthy individuals, identifying crucial strains such as *L. reuteri* and the key advantageous metabolite ICAld through culture-based screening. Through the development of a DLBCL xenograft mouse model, we continuously observed tumor development patterns and indicators of chemotherapy responses to thoroughly assess the sensitizing and toxicity-reducing impacts of *L. reuteri* and ICAld on CTX chemotherapy. Comprehensive research revealed that ICAld markedly enhanced the efficacy of CTX-triggered tumor cell destruction by suppressing excessive activation of the phosphatidylinositol 3-kinase (PI3K)/AKT/mechanistic target of rapamycin (mTOR) pathway and promoting the classical apoptotic pathway via mitochondrial outer membrane permeabilization. In addition, it stimulated AHR signaling, enhanced intestinal mucosal barrier function, reduced permeability, and mitigated chemotherapy-induced damage. This research paves the way to uncover the essential processes underlying “microbe–metabolite–host” dynamics in controlling DLBCL chemotherapy responsiveness, offering vital theoretical and experimental support for developing innovative approaches to tumor adjuvant treatment through microbiome alteration.

## Results

### The occurrence and progression of DLBCL are associated with reduced abundance of protective gut microbial species

To compare the gut microbiota composition between patients with DLBCL and healthy individuals, we enrolled a total of 21 newly diagnosed patients with DLBCL (D group) and 19 healthy volunteers (H group). Following collection from all participants, fecal samples were analyzed via full-length 16*S* ribosomal RNA (rRNA) gene (V1 to V9) sequencing. The groups were balanced at baseline with no significant differences in sex, age, or body mass index (see Tables S1 and S2 for full details).

**Fig. 1.**
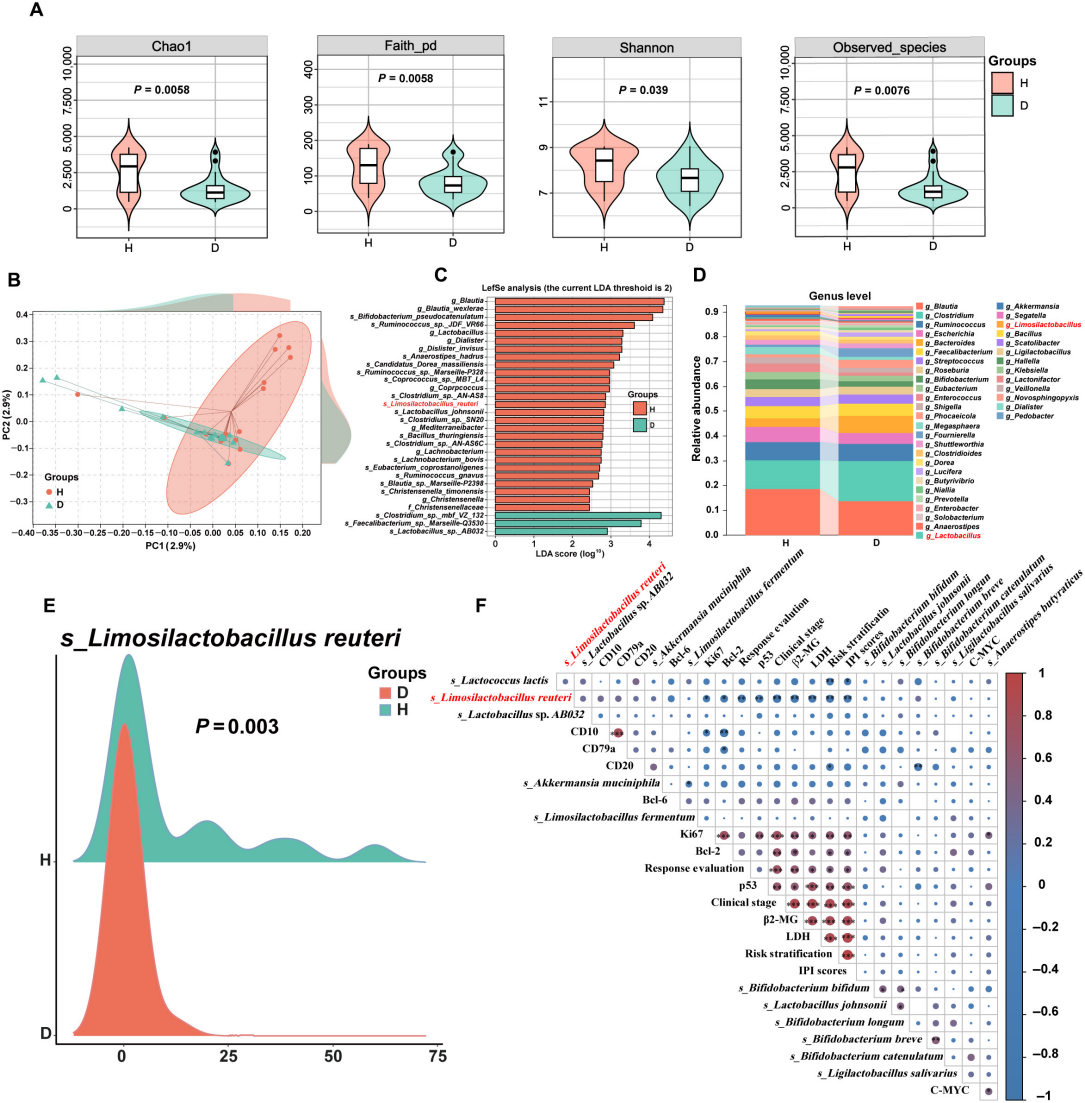
*L. reuteri* is a distinct bacterium in patients with diffuse large B cell lymphoma (DLBCL) compared with healthy individuals. (A) Within-sample (α) diversity of bacterial communities. (B) Between-sample (β) diversity in community structure. PC, principal component. (C) LDA effect size histogram of 16*S* ribosomal RNA (rRNA) sequences, showing significant taxa (LDA > 2). (D) Histogram of genus-level classification. (E) Ridge plot displaying the distribution of *L. reuteri* at the species level (*n* = 40). (F) Heatmap illustrating Spearman correlations between clinical traits and gut microbiota (blue, negative; red, positive). Data are means ± SD. Significance was assessed by one-way ANOVA (Dunnett’s) or Mann–Whitney *U* test, as appropriate. Correlations were analyzed using Spearman’s method. ns, not significant; *P < 0.05, **P < 0.01, and ***P < 0.001.

Assessment of α-diversity (Shannon and Chao1 indices) revealed that the gut microbiota in patients with DLBCL was significantly less diverse than that in healthy controls, and β-diversity analysis also showed that the composition of the gut microbiota in patients with DLBCL was distinct from that of the healthy control group (Fig. 1A and B). In addition, different microbial compositions were identified at both the species and genus levels (Fig. S1A to D). Linear discriminant analysis also revealed that several beneficial bacteria—including *L. reuteri*, *Lactobacillus johnsonii*, and *Bifidobacterium pseudocatenulatum*—had higher abundances in healthy individuals. Conversely, marked decreases were observed in patients with DLBCL (Fig. 1C to E). No significant differences were observed in the levels of nonbeneficial bacterial taxa (e.g., *Escherichia coli*, members of the phyla Actinobacteria and Bacteroidetes, and *Klebsiella* spp.), suggesting that the gut microbiota profile in DLBCL is primarily characterized by a reduction in beneficial commensals rather than an expansion of nonbeneficial taxa. This pattern was confirmed in an independent cohort of patients with natural killer T cell lymphoma. A significant negative correlation was found between *L. reuteri* levels and DLBCL disease burden, including risk stratification, clinical stage, International Prognostic Index (IPI) score, Ki67 index, and p53 mutation rate (Fig. 1F). Subgroup analysis showed that although there were no statistically significant differences in α- and β-diversity between germinal center B-cell-like (GCB) and activated B-cell-like subtypes, the genus *Limosilactobacillus* was significantly more abundant in the GCB subgroup, correlating with a better prognosis (Fig. S1E to G). These findings suggest that gut microbiota dysbiosis in patients with DLBCL is characterized by a loss of beneficial bacteria, with *L. reuteri* potentially playing a protective role during disease progression.

### Chemosensitization and toxicity reduction in DLBCL can be achieved through FMT from healthy donors

**Fig. 2.**
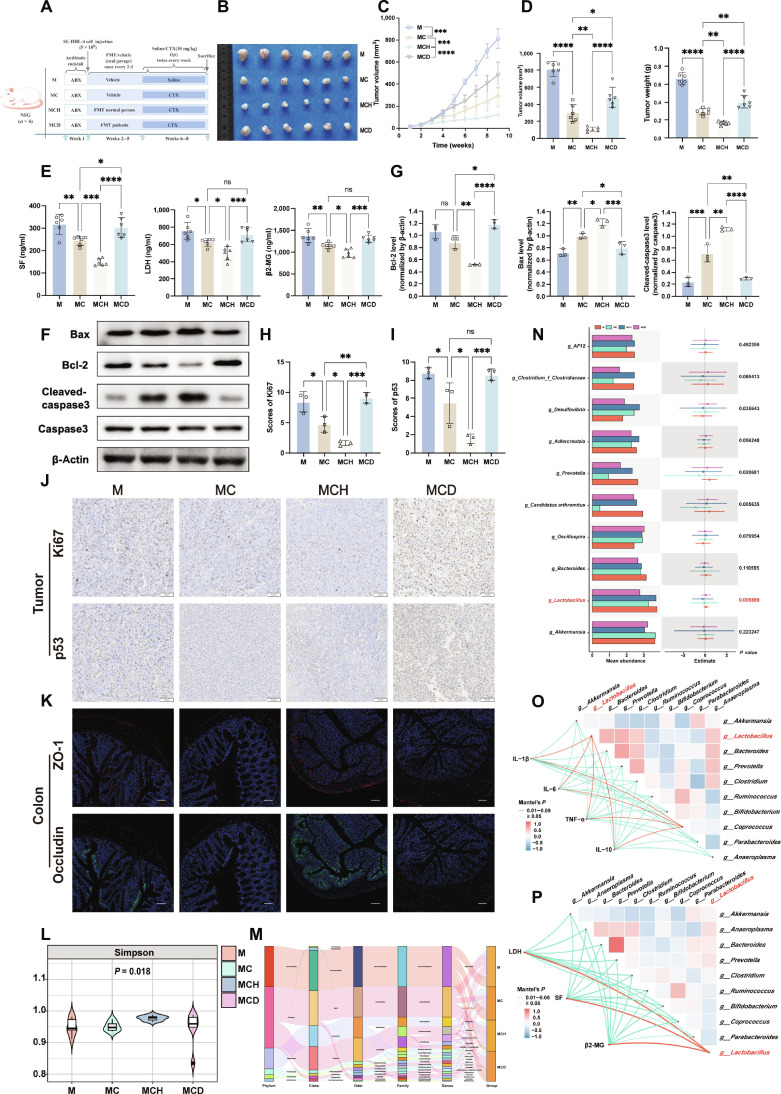
Fecal microbiota transplantation (FMT) modulates disease progression in diffuse large B cell lymphoma (DLBCL)-bearing mice. (A) Scheme of the mouse model: antibiotic (ABX) pretreatment, SU-DHL-4 cell inoculation, followed by fecal suspension gavage and cyclophosphamide (CTX) chemotherapy. ip, intraperitoneally. (B to D) Tumor phenotypes and representative image (B), growth curves (C), and endpoint volume/weight (D) (*n* = 6). (E) Serum tumor markers (β2-microglobulin [β2-MG], serum ferritin [SF], lactate dehydrogenase [LDH]; *n* = 6). (F to J) Molecular analyses in tumors: apoptosis proteins by Western blot (F and G) (*n* = 3) and Ki67/p53 expression by immunohistochemistry with quantification (H to J) (*n* = 3). Scale bars, 50 μm. (K) Gut barrier integrity assessed by immunofluorescence staining of zonula occludens-1 (ZO-1) and occludin in the colon. Scale bars, 50 μm. (L to N) Microbiome analysis: α-diversity (L), species composition (M), and differential species (N). (O and P) Correlation networks: microbiota abundance versus inflammatory cytokines (O) or serum tumor markers (P) (red, positive; blue, negative). Data are means ± SD. Significance was assessed by one-way ANOVA (Dunnett’s) or Mann–Whitney *U* test for comparisons and Spearman’s method for correlations. *P < 0.05, **P < 0.01, ***P < 0.001, and ****P < 0.0001; ns, not significant.

To investigate the role of gut microbiota in DLBCL progression and chemotherapeutic response, we established a DLBCL xenograft model in NSG (NOD-PrkdcscidIl2rgem1/Smo) mice. Following depletion of indigenous gut microbiota with broad-spectrum antibiotics, FMT was performed via oral gavage using donor specimens from healthy controls or patients with DLBCL. Selected experimental groups were subsequently treated with CTX (Fig. 2A). Compared to the model control group (M group), mice in the chemotherapy-only group (MC group) showed a significant reduction in tumor burden, including tumor weight, volume, and serum biomarkers (lactate dehydrogenase [LDH], serum ferritin [SF], and β2-microglobulin [β2-MG]), demonstrating that CTX effectively inhibited tumor growth (Fig. 2B to E). When combined with FMT from healthy donors (MCH group), CTX’s antitumor effect was further enhanced. Conversely, FMT from patients with DLBCL (MCD group) partially diminished CTX’s therapeutic efficacy. Western blot analysis revealed that the MCH group exhibited the most pronounced proapoptotic protein profile (including B cell lymphoma 2 [Bcl-2], Bax, and cleaved-caspase3) in tumor tissues, followed by the MC group. In contrast, the MCD group partially attenuated the proapoptotic effects of CTX (Fig. 2F and G). Immunohistochemistry results showed that the positivity rates for Ki67 and p53 were further decreased in the MCH group compared to the MC group (by 1.6% and 1.5%, respectively; *P* = 0.0461 and 0.0178). Conversely, these rates increased significantly in the MCD group compared with the MCH group (by 4.4%, *P* = 0.0002, and 3.1%, *P* = 0.0005), indicating higher tumor proliferative activity in the MCD group (Fig. 2H to J). These results suggest that FMT from healthy donors enhances the sensitivity of DLBCL to CTX, whereas FMT from patients reduces its effectiveness.

Since the gut is the primary site for microbiota-targeted interventions, we further assessed the effects of different treatments on intestinal health. We measured inflammatory cytokines (interleukin-1β [IL-1β], IL-6, IL-10, and tumor necrosis factor-α [TNF-α]) in colonic tissue and serum (Fig. S2A and B) and colon length (Fig. S2E and F) and performed Alcian blue periodic acid–Schiff (AB-PAS) staining of colon tissue (Fig. S2G). Although body weight did not differ significantly among the groups (Fig. S2C), the MCH group showed a restored colonic structure and less inflammation than the MC group. The MCD and M groups had fewer goblet cells, indicating impaired intestinal function, with more severe damage in the MCD group than in the MC group. Levels of intestinal tight junction proteins zonula occludens-1 (ZO-1) and occludin were higher in the MCH group but lower in the MCD group (Fig. 2K and Fig. S2H), suggesting that healthy donor FMT partially repaired the CTX-induced damage to the intestinal barrier.

Following antibiotic clearance, successful colonization of the humanized microbiota in the mouse gut was confirmed by 16*S* rRNA sequencing, revealing distinct microbial community structures among groups (Fig. S2K and L). Lower α- and β-diversity were observed in the MCD group compared to the MCH group (Fig. 2L and Fig. S2I). A significantly higher abundance of *Lactobacillus* was detected in the MCH group at both the genus and phylum levels (Fig. S2J, M, and N). Conversely, a decrease was observed in the MCD group (Fig. 2M and N), consistent with the trend seen in the clinical cohort. A negative correlation was identified between *Lactobacillus* abundance and both inflammatory and tumor burden (Fig. 2O and P and Fig. S2D). Overall, these findings suggest that gut microbiota composition significantly influences both the chemotherapeutic effectiveness of CTX against DLBCL and its intestinal toxicity. Chemosensitivity was improved by healthy donor FMT through remodeling the gut microbiota, enhancing barrier function, and reducing inflammation. Conversely, patient-derived FMT disrupted gut homeostasis and decreased treatment efficacy, with *Lactobacillus* appearing to play a key role.

### *L. reuteri* HG001 identified as a key probiotic strain improving DLBCL chemotherapy effectiveness

To identify key bacterial strains with therapeutic potential from healthy human guts, we used culturomics to isolate *L. reuteri* HG001, *L. johnsonii*, and *B. pseudocatenulatum*. Their inhibitory effects on DLBCL cell lines (SU-DHL-4 and OCI-LY3) were compared. Results showed that *L. reuteri* HG001 had the most significant impact on promoting early apoptosis and depolarizing mitochondrial membrane potential in tumor cells (Fig. S3-1A and B). Further assessment of its probiotic properties (Fig. S3-2A to J) indicated that this short-rod bacterium shows strong resistance to antibiotics such as ciprofloxacin and cotrimoxazole, inhibits pathogens such as *E. coli* and *Staphylococcus aureus*, and demonstrates good tolerance to gastric acid and bile salts, along with antioxidant activity, supporting its potential *in vivo* function.

**Fig. 3.**
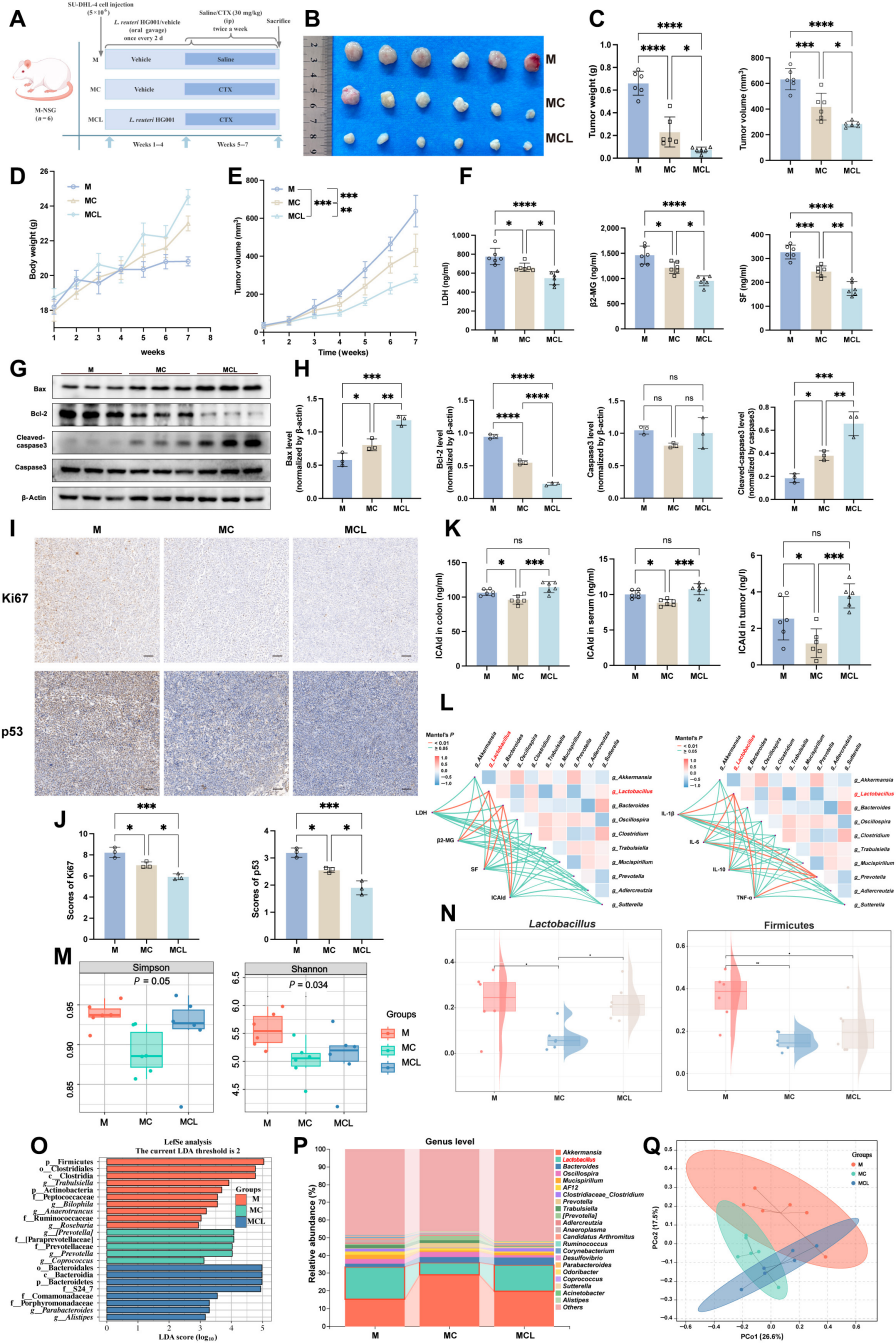
Oral gavage of *L. reuteri* HG001*.* Mucosae regulate disease progression in diffuse large B cell lymphoma (DLBCL)-bearing mice. (A) Experimental scheme: NSG (NOD-PrkdcscidIl2rgem1/Smo) mice were subcutaneously injected with SU-DHL-4 cells (5 × 10^6^) and treated with twice-daily oral gavage of *L. reuteri* HG001 (1 × 10^9^ colony-forming units [CFU]/ml in gelatin saline) plus intraperitoneal cyclophosphamide (CTX) (30 mg/kg, twice weekly, from week 5). (B) Representative DLBCL tumors across groups (*n* = 6). (C and E) Tumor volume dynamics (E) and final volume/weight (C) (*n* = 6). (D) Mouse body weight changes (*n* = 6). (F) Serum levels of β2-microglobulin (β2-MG), serum ferritin (SF), and lactate dehydrogenase (LDH) (*n* = 6). (G and H) Western blot analysis of apoptosis markers (Bax, B cell lymphoma 2 [Bcl-2], and cleaved-caspase3) in tumors (*n* = 3). (I and J) Representative immunohistochemical images and quantification for Ki67 and p53 in tumors (*n* = 3). Scale bars, 50 μm. (K) Indole-3-carbaldehyde (ICAld) concentrations in colon, serum, and tumors (*n* = 6). (L) Correlation network heatmap of inflammatory cytokines, ICAld, DLBCL serum markers, and gut microbiota abundance (red, positive; blue, negative). (M) Microbial α-diversity. (N) Cloud-rain plot of *Lactobacillus*, Actinobacteria, and Firmicutes relative abundance. (O) Bar chart of differentially abundant bacteria (LDA > 2). (P) Genus-level composition histogram. (Q) β-Diversity (microbial community composition differences). Data are means ± SD. Significance was determined by one-way ANOVA (Dunnett’s) or Mann–Whitney *U* test; correlations were analyzed using Spearman’s method. *P < 0.05, **P < 0.01, ***P < 0.001, and ****P < 0.0001; ns, not significant.

To assess the *in vivo* antitumor effects of *L. reuteri* HG001, we orally gavaged DLBCL-bearing mice with live bacteria in combination with CTX treatment (Fig. 3A). The live bacterial intervention did not affect overall mouse growth, indicating that it is safe. Compared to CTX alone (MC group), the combination with *L. reuteri* HG001 (MCL group) more effectively suppressed tumor growth, as shown by reduced tumor volume (*P* = 0.0225) and weight (*P* = 0.0357), slower growth (Fig. 3B to E), and lowered serum markers linked to tumor burden, such as LDH, SF, and β2-MG (Fig. 3F). Western blot analysis showed increased levels of proapoptotic proteins Bax and cleaved-caspase3, and decreased Bcl-2 in the MCL group (Fig. 3G and H). Immunohistochemistry indicated that the positive rates of Ki67 and p53 in the MCL group decreased by 1.1% (*P* = 0.0226) and 0.7% (*P* = 0.0119), respectively, compared to the MC group (Fig. 3I and J); this suggests that the bacteria work synergistically with CTX to promote apoptosis and inhibit cell proliferation. In addition, *L. reuteri* HG001 intervention significantly increased ICAld concentrations in mice. Compared with the chemotherapy group (MC group), ICAld levels in the colon tissue, serum, and tumor tissue were significantly elevated in the *L. reuteri* HG001 combination intervention group (MCL group) (colon: 114.32 ± 7.80 ng/ml versus 96.14 ± 6.50 ng/ml; serum: 10.74 ± 0.78 ng/ml versus 8.86 ± 0.45 ng/ml; tumor: 3.78 ± 0.70 ng/l versus 1.19 ± 0.28 ng/l), with all differences being statistically significant (all *P* < 0.05) (Fig. 3K).

Regarding intestinal effects, oral gavage of *L. reuteri* HG001 reduced colonic inflammation and enhanced barrier integrity, as shown by increased colon length (*P* < 0.001), a more favorable pro-/anti-inflammatory cytokine ratio (Fig. S3-3A to C), higher goblet cell counts, and more intact tissue morphology (Fig. S3-3F). 16*S* rRNA sequencing indicated that the α- and β-diversity of the gut microbiota in the MCL group were higher than in the MC group, with increased levels of the genus *Lactobacillus* and the phylum Firmicutes (Fig. S3-3G to J). The abundance of *Lactobacillus* was negatively correlated with both inflammatory and tumor burden (Fig. 3L to Q and Fig. S3-3D and E). These findings suggest that *L. reuteri* HG001 is a key beneficial bacterium that enhances the anti-DLBCL effects of CTX and reduces its intestinal toxicity.

### Suppression of DLBCL cell proliferation mainly mediated by ICAld from *L. reuteri* HG001

To identify the active antitumor components of *L. reuteri* HG001, we first measured the median inhibitory concentration of CTX and the inhibitory effects of various bacterial components—live bacteria, heat-inactivated bacteria, and supernatant (scanning electron microscopy images of live and heat-inactivated bacteria are shown in Fig. S4-2A)—on DLBCL cell lines. CTX was cocultured with the cells for 20 h, followed by the addition of bacterial components for another 4 h to evaluate their effects on proliferation and apoptosis (Fig. S4-1A to E). The results showed that both live bacteria and the supernatant significantly inhibited proliferation and induced apoptosis in a time-dependent manner. In contrast, heat-killed bacteria had no significant effect (Fig. S4-1F to L), indicating that the antitumor activity mainly derives from metabolites in the supernatant. Further targeted liquid chromatography-tandem mass spectrometry (LC–MS/MS) metabolomic analysis revealed significantly increased levels of tryptophan metabolites in the supernatant, particularly ICAld and indole-3-lactic acid (ILA) (Fig. S4-1M and 2H). Analysis of clinical samples showed that serum levels of ICAld, ILA, and xanthurenic acid (XA) were lower in patients with DLBCL than in healthy controls. In addition, oral gavage of live bacteria increased fecal levels of ICAld and ILA in tumor-bearing mice (Fig. 3K and Fig. S4-1O, P, and R), suggesting that tryptophan metabolites may mediate the antitumor effects.

**Fig. 4.**
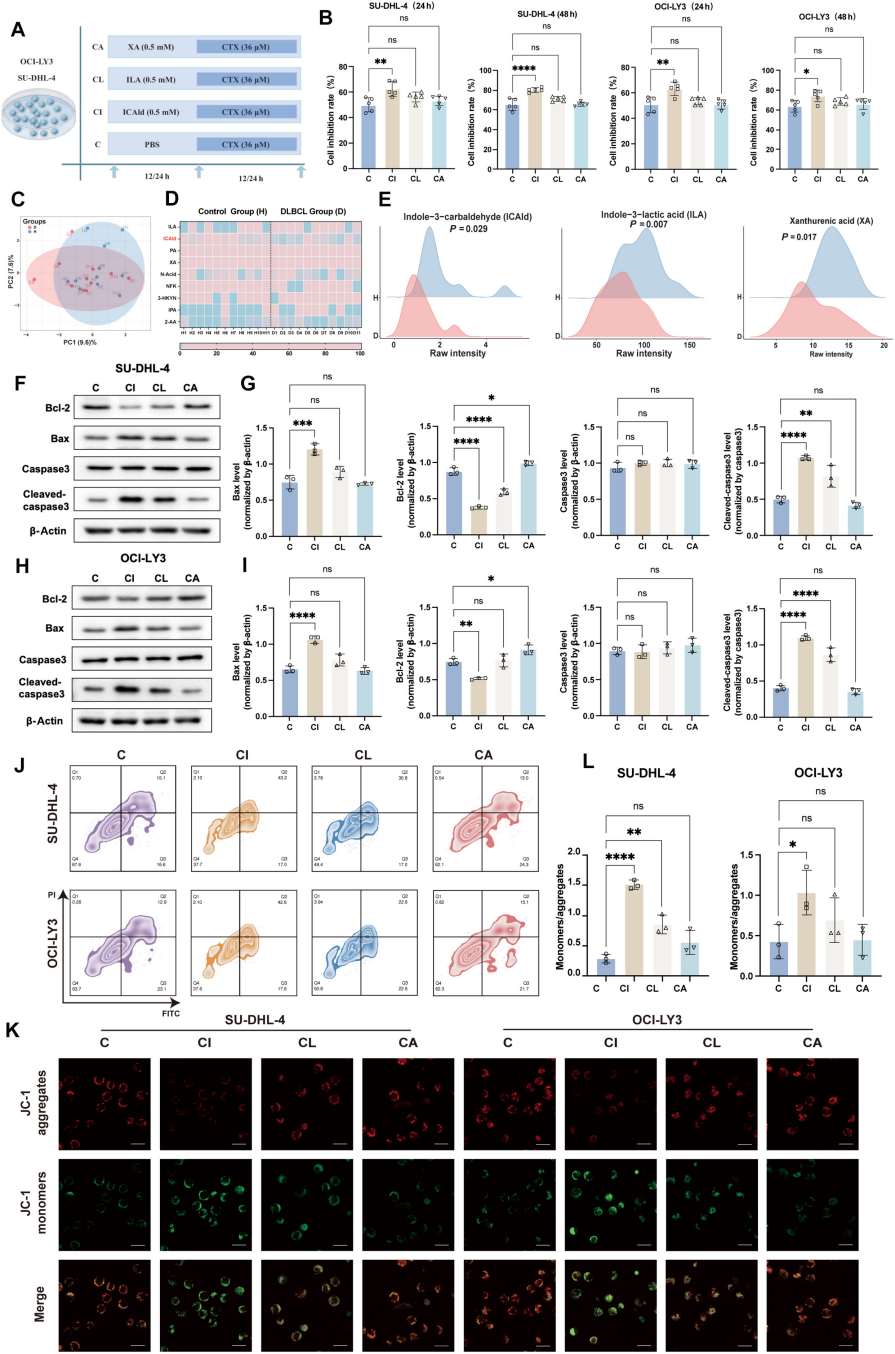
Indole-3-carbaldehyde (ICAld) is the primary beneficial metabolite of *L. reuteri* HG001. in synergy with cyclophosphamide (CTX) for anti-diffuse large B cell lymphoma (DLBCL) activity. (A) Experimental design: coincubation of ICAld, indole-3-lactic acid (ILA), and xanthurenic acid (XA) with DLBCL cell lines (SU-DHL-4 and OCI-LY3) for 12/24 h (all at 0.5 mM concentration), followed by additional intervention with CTX (36 μM) for 12/24h. (B) Inhibition rates of 2 DLBCL cell lines (SU-DHL-4 and OCI-LY3) at 24 and 48h postintervention, as determined by Cell Counting Kit-8 (CCK-8) assay. (C) Principal components analysis of metabolic composition differences. (D) Heatmap of targeted tryptophan metabolomics in human serum. (E) Ridge plot depicting relative serum concentrations of ICAld, ILA, and XA. (F to I) Protein expression and quantification of apoptotic markers (Bax, B cell lymphoma 2 [Bcl-2], and cleaved-caspase3) in treated SU-DHL-4 and OCI-LY3 cells (*n* = 3). (J) Flow cytometric measurement of apoptosis. (K) JC-1 staining images showing mitochondrial membrane potential. Scale bars, 20 μm. (L) Quantification of JC-1 signal ratio (*n* = 3). Data were analyzed by one-way ANOVA (Dunnett’s) or Mann–Whitney *U* test. *P < 0.05, **P < 0.01, ***P < 0.001, and ****P < 0.0001; ns, not significant.

To identify the key active metabolite, we treated DLBCL cell lines with various concentrations of ICAld, XA, and ILA for 24 and 48 h (Fig. S4-2B to F). ICAld showed effects similar to those of bacterial supernatant, inhibiting proliferation and inducing apoptosis in a time- and dose-dependent manner, especially the ICAld (Fig. S4-3A and B). It increased the expression of proapoptotic proteins, such as Bax and cleaved-caspase3, and depolarized mitochondrial membrane potential. When combined with CTX, the CTX + ICAld (CI) group exhibited the most potent inhibition of proliferation (Fig. 4A to E), the most significant mitochondrial membrane potential depolarization, and the highest apoptosis rate. The CTX + ILA (CL) group ranked second, while the CTX + XA (CA) group showed no notable synergistic effect. Western blot analysis confirmed that the CI group had the highest expression of proapoptotic proteins (Fig. 4F to I). The results from the mitochondrial membrane potential assay and the flow cytometric apoptosis detection were consistent, showing the same trend (Fig. 4J to L). These findings suggest that ICAld is one of the primary active metabolite responsible for *L. reuteri* HG001’s antitumor activity.

### The inhibitory effect of ICAld on DLBCL stems from the tryptophan metabolic pathway of *L. reuteri* HG001

**Fig. 5.**
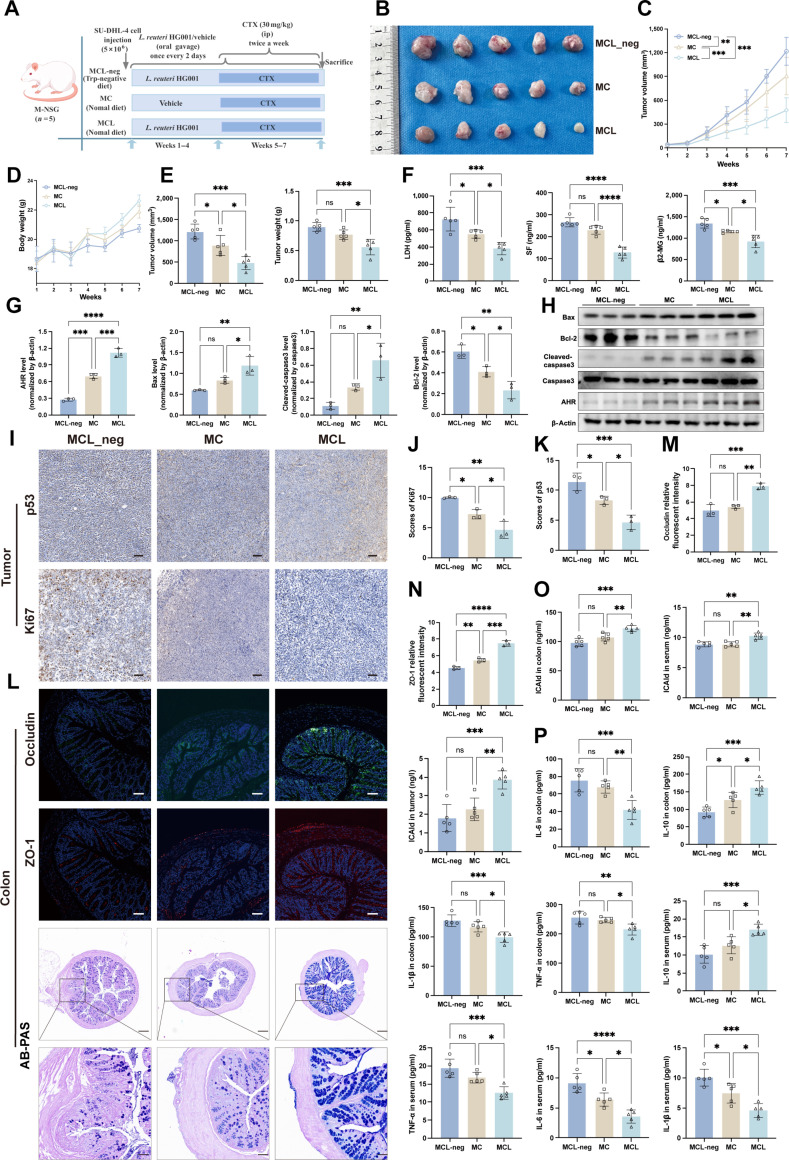
A tryptophan-deficient diet reverses the synergistic antitumor effects of *L. reuteri* HG001*.* in diffuse large B cell lymphoma (DLBCL)-bearing mice. (A) Experimental design: NSG (NOD-PrkdcscidIl2rgem1/Smo) mice were inoculated with a DLBCL tumor model and orally administered *L. reuteri* HG001 (100 μl, 1 × 10^9^ colony-forming units [CFU]/ml, every 2 days for 7 weeks). Starting from week 5, cyclophosphamide (CTX) (30 mg/kg, twice weekly for 3 weeks) was administered via intraperitoneal injection. The MCL_neg group received a tryptophan-deficient diet, while the remaining mice were fed a standard diet. (B) Visual representation of DLBCL tumors across different groups (*n* = 5). (C) Changes in DLBCL tumor volume over time (*n* = 5). (D) Alterations in mouse body weight (*n* = 5). (E) Tumor volume and weight at the conclusion of the intervention (*n* = 5). (F) Serum levels of β2-microglobulin (β2-MG), serum ferritin (SF), and lactate dehydrogenase (LDH) (*n* = 5). (G and H) Representative Western blot images and quantitative bar charts (*n* = 3) evaluating aryl hydrocarbon receptor (AHR) and apoptosis-related signaling molecules (Bax, B cell lymphoma 2 [Bcl-2], cleaved-caspase3) in DLBCL tumor tissues. (I to K) Representative immunohistochemical staining images (scale bars, 50 μm) and quantitative bar charts (*n* = 3) for Ki67 and p53 expression in DLBCL tumor tissues. (L to N) Representative immunofluorescence staining images (scale bars, 50 μm) for zonula occludens-1 (ZO-1) and occludin, along with Alcian blue periodic acid–Schiff (AB-PAS) staining and quantitative bar charts (*n* = 3) in colonic tissues. Scale bars, 50 μm (top) and 20 μm (bottom). (O) Relative concentrations of indole-3-carbaldehyde (ICAld) in colonic tissues, serum, and DLBCL tumors (*n* = 5). (P) Inflammatory cytokine (interleukin-1β [IL-1β], IL-6, IL-10, and tumor necrosis factor-α [TNF-α]) levels in both serum and colon tissue; statistical analysis by one-way ANOVA (Dunnett’s) or Mann–Whitney *U* test. Significance: *P < 0.05, **P < 0.01, ***P < 0.001, and ****P < 0.0001; ns, not significant.

Since *L. reuteri* HG001metabolizes dietary tryptophan into the AHR ligand ICAld, it was investigated whether dietary tryptophan levels affect the antitumor response mediated by this bacterium. To do this, we assigned mice to either a normal tryptophan (MCL) or a tryptophan-deficient (MCL_neg) diet, which were maintained throughout the experiment (Fig. 5A). Although the tumor-suppressing effect of *L. reuteri* HG001 was not completely lost with the low-tryptophan (0.19%) diet, significantly more tumor suppression was seen in mice treated with *L. reuteri* HG001 and fed a high-tryptophan (1.19%) diet compared to those on the tryptophan-deficient diet. The MCL_neg group showed increased tumor burden and higher levels of inflammation compared to the MCL group, along with up-regulated antiapoptotic protein expression (Fig. 5B to H and Fig. S5D), and higher proportions of Ki67- and p53-positive cells by 5.37% (*P* = 0.0011) and 6.72% (*P* = 0.001), respectively (Fig. 5I to K). Metabolomic analysis revealed lower ICAld levels in the MCL_neg group, and its concentration was positively correlated with tryptophan intake.

Regarding intestinal function, the MCL_neg group exhibited significantly shorter colon length (*P* = 0.003) (Fig. S5A and B) and decreased expression of tight junction proteins ZO-1 (*P* < 0.001) and occludin (*P* = 0.0008). It compromised colonic mucosal integrity, with fewer goblet cells as shown by ZO-1, occludin, and AB-PAS staining (Fig. 5L to N). Under a tryptophan-deficient diet, the level of ICAld was significantly reduced in tumor-bearing mice gavaged with *L. reuteri* HG001 and was markedly lower than that in the normal diet group, and the same pattern was observed for the levels of inflammatory factors (Fig. 5O and P). Correlation analysis indicated that ICAld levels were negatively associated with both proinflammatory factors and tumor burden markers (Fig. S5C). Overall, these findings confirm that *L. reuteri* HG001 inhibits DLBCL through ICAld derived from its tryptophan metabolism; tryptophan deficiency reverses this protective effect and exacerbates intestinal barrier damage.

### ICAld suppresses disease progression in DLBCL-bearing mice in a concentration-dependent manner

**Fig. 6.**
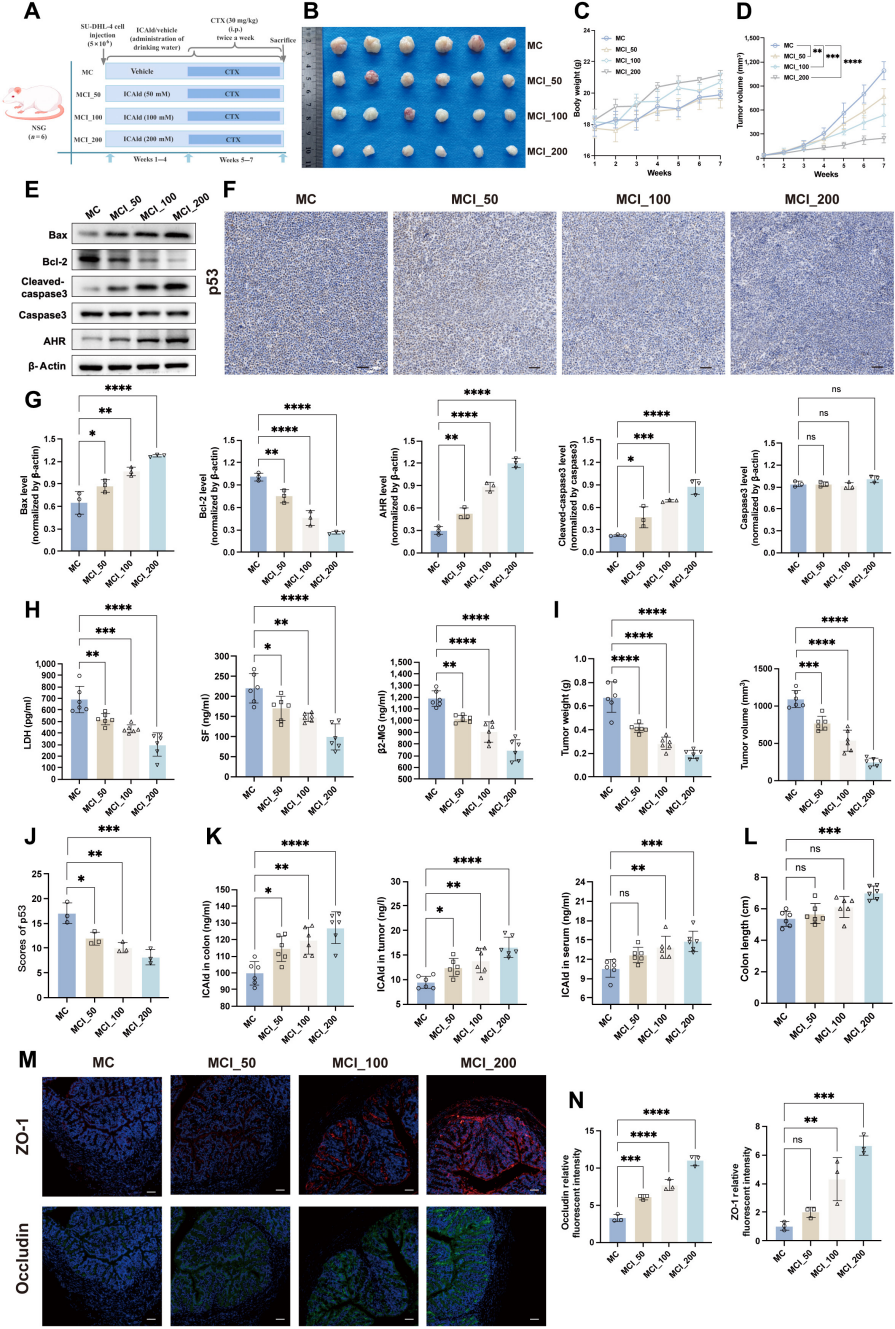
Indole-3-carbaldehyde (ICAld) synergizes with cyclophosphamide (CTX) to delay diffuse large B cell lymphoma (DLBCL) progression in a concentration-dependent manner. (A) Experimental design: DLBCL-bearing mice were administered varying concentrations of ICAld (50, 100, and 200 mM) via drinking water for 7 weeks, with CTX (30 mg/kg, twice weekly for 3 weeks) introduced starting from week 5. (B) Visual representation of DLBCL tumors across different groups (*n* = 6). (C) Alterations in mouse body weight (*n* = 6). (D) Changes in DLBCL tumor volume (*n* = 6). (E) Representative Western blot images in DLBCL tumor tissues. (F) Representative immunohistochemical staining images (scale bars, 50 μm) for p53 expression in DLBCL tumor tissues. (G) Representative Western blot images and quantitative bar charts (*n* = 3) evaluating aryl hydrocarbon receptor (AHR) and apoptosis-related signaling molecules (Bax, B cell lymphoma 2 [Bcl-2], cleaved-caspase3) in DLBCL tumor tissues. (H) Serum levels of β2-microglobulin (β2-MG), serum ferritin (SF), and lactate dehydrogenase (LDH) (*n* = 6). (I) Tumor weight and tumor volume at the conclusion of the intervention (*n* = 6). (J) Quantitative bar charts (*n* = 3) for p53 expression in DLBCL tumor tissues. (K) ICAld concentrations in colon, serum, and tumor tissues. (L) Group-wise colon length (*n* = 6). (M and N) Zonula occludens-1 (ZO-1) and occludin immunofluorescence: images (scale bars, 50 μm) and quantification (*n* = 3). Significance was assessed by one-way ANOVA (Dunnett’s) or Mann–Whitney *U* test for comparisons and Spearman’s method for correlations. *P < 0.05, **P < 0.01, ***P < 0.001, and ****P < 0.0001; ns, not significant.

To evaluate the *in vivo* antitumor potential of ICAld, we gave DLBCL-bearing mice 50, 100, or 200 mM ICAld in their drinking water, while all other treatments remained the same (Fig. 6A). Compared to the control group (MC group), ICAld combination therapy significantly reduced tumor burden and alleviated the inflammatory status *in vivo* (Fig. 6B to J and Fig. S6-1A and B), as shown by smaller tumor volume and weight, increased expression of proapoptotic proteins Bax and cleaved-caspase3, decreased Bcl-2 levels, and a lower percentage of p53-positive cells. The antitumor effect of ICAld depended on the concentration, with the highest dose group (MCI_200) showing the best efficacy. Serum ICAld levels matched the administered concentrations (Fig. 6K) and were inversely related to tumor burden. In addition, ICAld treatment improved colonic barrier structure and reduced inflammation. The MCI_200 group had the longest colon, the most goblet cells, the least inflammation, and the strongest barrier integrity (Fig. 6L to N), indicating that ICAld helps reduce chemotherapy-related damage to the intestinal microenvironment and barrier function. Correlation analyses—the *in vivo* concentration of ICAld showed a significant positive correlation with colon length and the immunofluorescence intensity of ZO-1/occludin in colon tissue—supported this finding (Fig. S6-2C).

To further evaluate potential toxicity, we conducted histopathological examinations of major organs using hematoxylin and eosin (HE) staining after the 7-week intervention. Results showed preserved tissue architecture in the heart, liver, spleen, lungs, and kidneys across all dose groups, with no significant inflammation or necrosis observed (Fig. S6-1C). Serum biochemical parameters and complete blood count analysis indicated no cardiotoxicity, nephrotoxicity, or hematologic toxicity (Fig. S6-1D and 2A and B), with all values staying within the reference ranges of the MC group. These findings suggest that short-term administration of 50 to 200 mM ICAld does not cause significant organ toxicity, although its long-term safety remains to be further validated.

### ICAld mediates suppression of DLBCL cell proliferation through AHR activation and mTOR pathway inhibition

**Fig. 7.**
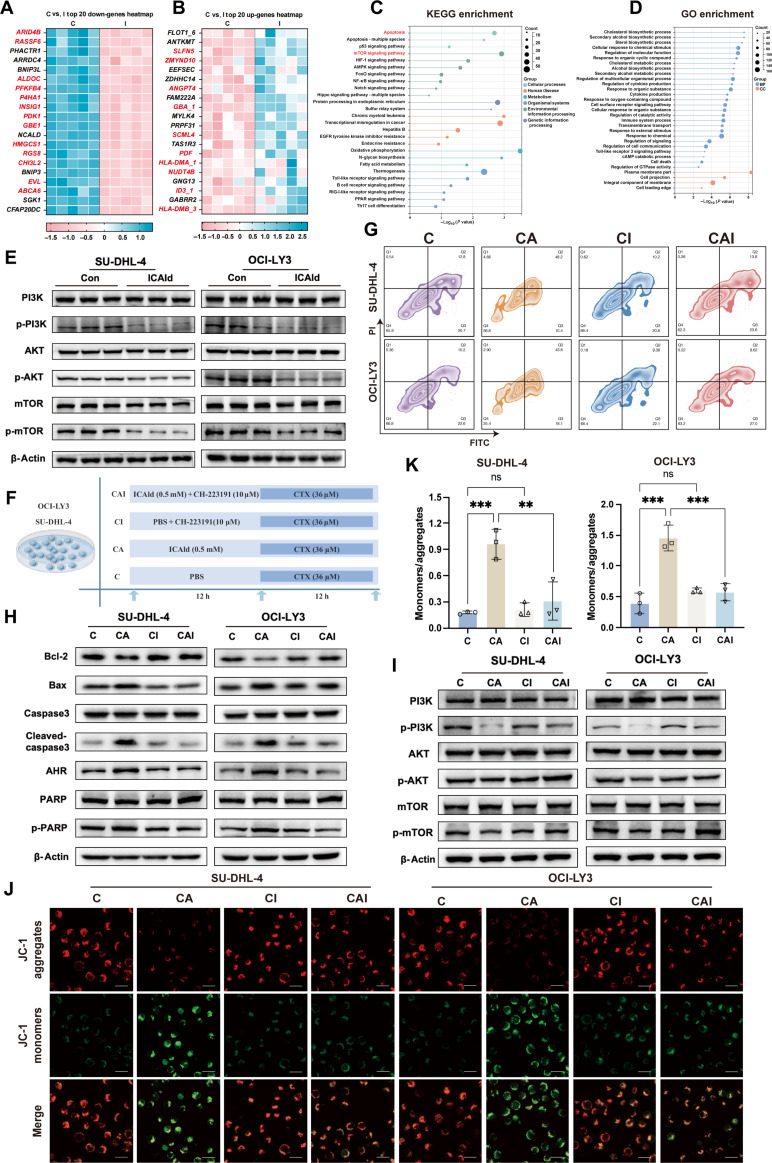
Indole-3-carbaldehyde (ICAld) activates aryl hydrocarbon receptor (AHR) to suppress phosphatidylinositol 3-kinase (PI3K)/AKT/mechanistic target of rapamycin (mTOR) signaling pathway activation, promote apoptosis initiation, and enhance cyclophosphamide (CTX)-induced cytotoxicity in diffuse large B cell lymphoma (DLBCL) cells. (A and B) Heatmaps display differentially expressed genes in SU-DHL-4 cells treated with ICAld versus phosphate-buffered saline (PBS) control. (C and D) Lollipop plots summarize Gene Ontology (GO) and Kyoto Encyclopedia of Genes and Genomes (KEGG) enrichment analyses. AMPK, adenosine monophosphate kinase; EGFR, epidermal growth factor receptor; PPAR, peroxisome proliferator–activated receptor; Th17, T helper 17; cAMP, cyclic adenosine monophosphate; GTPase, guanosine triphosphatase. (E, H, and I) Western blot analysis evaluates the expression of AHR, phospho-proteins in the PI3K/AKT/mTOR pathway (p-PI3K, p-AKT, and p-mTOR), and apoptosis-related markers (Bax, B cell lymphoma 2 [Bcl-2], cleaved-caspase3, and p-PARP) in DLBCL cell lines (SU-DHL-4 and OCI-LY3) following ICAld treatment. (F) Experimental scheme for co-treatment with ICAld (0.5 mM), AHR antagonist CH-223191 (10 μM), and CTX (36 μM). (G) Apoptosis rates quantified by flow cytometry. (J and K) Schematic and quantitative analysis (*n* = 3) of mitochondrial membrane potential changes (JC-1 assay) in cells treated with ICAld ± CH-223191. Scale bar, 20 μm. Statistical significance was determined by one-way ANOVA (Dunnett’s) or Mann–Whitney *U* test. **P < 0.01, and ***P < 0.001; ns, not significant.

To clarify the molecular mechanism by which ICAld inhibits DLBCL, we performed RNA sequencing on SU-DHL-4 cells treated with ICAld. The results showed changes in genes related to apoptosis and oxidative stress, which were among the most significantly differentially expressed genes (Fig. 7A and B). Gene Ontology (GO) and Kyoto Encyclopedia of Genes and Genomes (KEGG) enrichment analyses indicated increased apoptosis and reduced proliferation after ICAld treatment, with notable enrichment in the mTOR signaling and apoptosis pathways (Fig. 7C and D). Western blot analysis revealed that ICAld decreased the phosphorylation of p-PI3K, p-AKT, and p-mTOR in 2 DLBCL cell lines and increased phosphatase and tensin homolog (PTEN) expression. At the same time, levels of proapoptotic proteins Bax, cleaved-caspase3, and phosphorylated poly(ADP-ribose) polymerase (p-PARP) rose, while Bcl-2 levels dropped (Fig. 7E and Fig. S7-1A).

Recent studies suggest that ICAld derived from *L. reuteri* HG001 can function as an endogenous ligand for the AHR. Molecular docking confirmed a favorable binding interaction between ICAld and AHR (Fig. S7-1D and E). Cytochrome P450 family 1 subfamily A member 1 (CYP1A1), a key downstream effector of AHR, belongs to the same P450 family as CYP3A4, a crucial enzyme for CTX metabolism. The ROS generated by CYP1A1 during oxidative metabolism may synergize with ROS produced during CTX metabolism, collectively inhibiting the PI3K/AKT/mTOR pathway and activating mitochondrial apoptosis. To validate whether ICAld regulates the mTOR pathway and apoptosis via the AHR/CYP1A1/oxidative stress axis, we used the AHR antagonist CH-223191. In the antagonist group (ICAld_I), AHR expression was down-regulated, PTEN expression decreased, and the levels of p-PI3K, p-AKT, and p-mTOR rebounded (Fig. S7-1F and G), indicating that ICAld’s inhibition of the PI3K/AKT/mTOR pathway is dependent on AHR activation.

However, whether ICAld exerts selective cytotoxicity against DLBCL cells—specifically, whether this metabolite preferentially targets tumor cells while sparing normal rapidly dividing cells—remains unclear. To address this, we compared the effects of ICAld at the same concentration (0.5 mM) on ROS generation in DLBCL cells (SU-DHL-4) versus normal B lymphocytes (GM12878), as well as in colon adenocarcinoma cells (HT29) versus normal colon epithelial cells (NCM460), using flow cytometry (Fig. S7-2A and B). The results demonstrated that ICAld induced a significantly stronger pro-oxidative effect in tumor cells than in normal cells, with no marked increase in ROS levels observed in normal cells. Furthermore, the impact of ICAld on cell viability was assessed using the Cell Counting Kit-8 (CCK-8) assay, which revealed a more pronounced inhibitory effect on the proliferation of tumor cells (SU-DHL-4 and HT29) compared to normal cells (GM12878 and NCM460) (Fig. S7-2C). Collectively, these findings indicate that ICAld exhibits selective cytotoxicity toward DLBCL and other tumor cells, suggesting a favorable safety profile for its potential application in DLBCL therapy.

Furthermore, when ICAld was combined with CTX treatment, an AHR antagonist (CAI) group was included. The combined intervention group (CA group) exhibited increased AHR expression, a higher rate of apoptosis, and inhibition of phosphorylation of the PI3K/AKT/mTOR signaling pathway. In contrast, these effects were reversed in the CAI group (Fig. 7F to I and Fig. S7-1B and C). The JC-1 assay showed greater mitochondrial membrane potential depolarization in the CA group than in the control, as evidenced by a lower green/red fluorescence ratio (0.79, *P* = 0.0007). No significant difference was found between the CAI group and the CI group (CTX + AHR antagonist) (Fig. 7J and K), ruling out nonspecific interference. Molecular docking simulations indicated that both the p85α and p110α subunits of PI3K can bind to AHR (Fig. S8-2B and C). These findings demonstrate that ICAld exerts its anti-DLBCL effects by activating AHR, inhibiting the PI3K/AKT/mTOR pathway, and promoting mitochondrial apoptosis.

### Chemosensitizing effects of *L. reuteri* HG001 and ICAld on DLBCL can be reversed by AHR antagonist

**Fig. 8.**
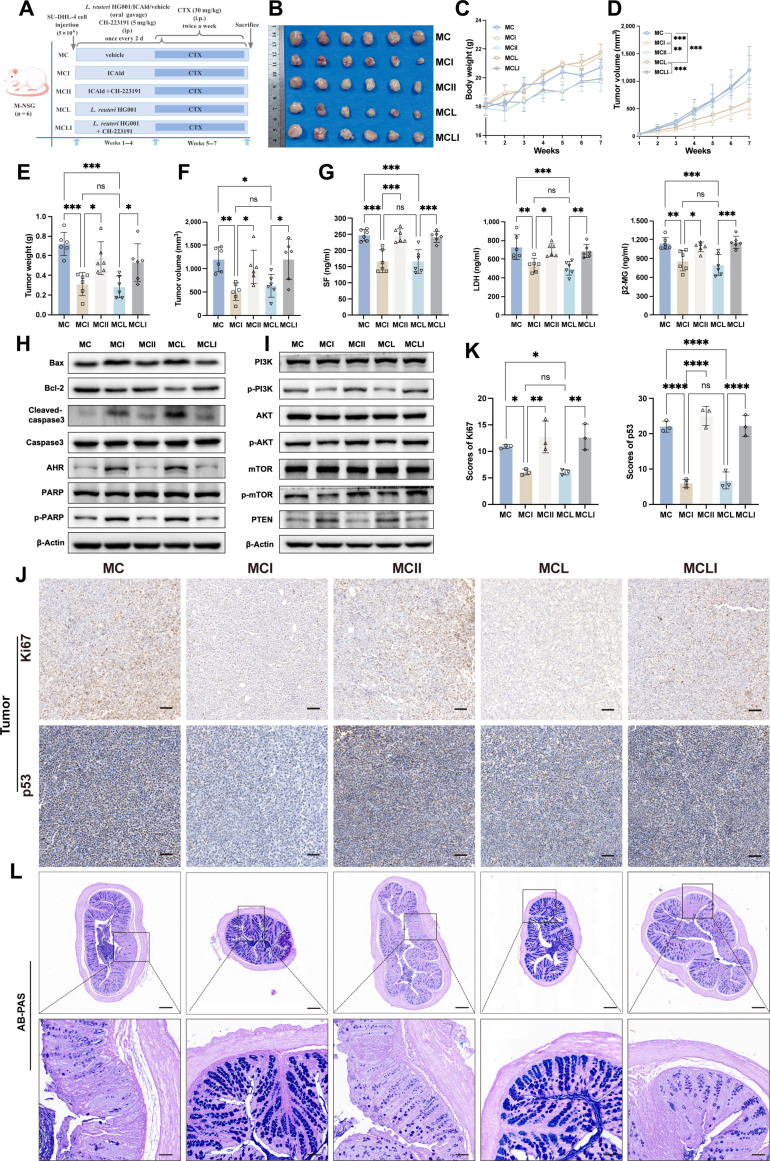
Aryl hydrocarbon receptor (AHR) antagonists reverse the synergistic anti-diffuse large B cell lymphoma (DLBCL) effects of *L. reuteri* HG001 and indole-3-carbaldehyde (ICAld) in combination with cyclophosphamide (CTX). (A) Experimental design: NSG (NOD-PrkdcscidIl2rgem1/Smo) mice were subcutaneously injected with SU-DHL-4 cells (5 × 10^6^ cells/100 μl) to establish a tumor-bearing model. Mice received oral gavage of ICAld and *L. reuteri* HG001 (once every other day for 4 weeks, at previously specified doses), with experimental groups additionally administered the AHR antagonist CH-223191 (intraperitoneal injection, 5 mg/kg) for 7 weeks. CTX treatment (intraperitoneal injection, 30 mg/kg, twice weekly for 3 weeks) commenced in week 5. (B) Visual representation of DLBCL tumors across different groups following the intervention (*n* = 6). (C and D) Body weight and tumor volume changes (*n* = 6). (E and F) Tumor weight and tumor volume at the conclusion of the intervention (*n* = 6). (G) Hematological parameters indicative of tumor burden (lactate dehydrogenase [LDH], serum ferritin [SF], β2-microglobulin [β2-MG]). (H and I) Representative Western blot images evaluating AHR protein, phosphatidylinositol 3-kinase (PI3K)-AKT–mechanistic target of rapamycin (mTOR) pathway components (p-PI3K, p-AKT, p-mTOR, and phosphatase and tensin homolog [PTEN]), and apoptosis-related signaling molecules (Bax, B cell lymphoma 2 [Bcl-2], cleaved-caspase3, and p-poly[adenosine 5′-diphosphate-ribose] polymerase [PARP]) in DLBCL tumor tissues. (J and K) Representative immunohistochemical staining images (scale bars, 50 μm) and quantitative analysis results (*n* = 3) for Ki67 and p53 expression in DLBCL tumor tissues. (L) Schematic representation of Alcian blue periodic acid–Schiff (AB-PAS) staining in colonic tissues. Scale bars, 50 μm (top) and 20 μm (bottom). Statistical significance was determined using one-way ANOVA with Dunnett’s multiple comparisons test and Mann–Whitney *U* test for intergroup comparisons. ns, not significant; *P < 0.05, **P < 0.01, ***P < 0.001, and ****P < 0.0001.

Finally, *in vivo* experiments were conducted to confirm the mechanism by which ICAld works in conjunction with CTX against DLBCL. As shown in Fig. 8A, tumor-bearing mice were treated either by oral gavage with *L. reuteri* HG001 or by ICAld in drinking water, along with an AHR antagonist control group. Compared to the MC group, treatments with either ICAld (MCI group) or *L. reuteri* HG001 (MCL group) resulted in decreased tumor size, as indicated by smaller tumor volume and weight, slower tumor growth, and lower levels of serum biomarkers such as LDH, SF, and β2-MG (Fig. 8B to G). Western blot results showed that tumor tissues from the MCI and MCL groups had higher levels of AHR and proapoptotic proteins (Bax, cleaved-caspase3, and p-PARP), lower Bcl-2 levels, reduced phosphorylation of the PI3K/AKT/mTOR pathway, and increased PTEN levels (Fig. 8H and I and Fig. S8-1B). The similar effects on phenotype by *L. reuteri* HG001 and ICAld suggest that ICAld is the primary active metabolite. Intervention with AHR antagonists (MCII and MCLI groups) increased the proportions of Ki67- and p53-positive cells in tumor tissues (Fig. 8J and K).

Further analysis showed that systemic inflammation levels decreased in the MCI and MCL groups (Fig. S8-2A), an effect reversed by the AHR antagonist. Colonic AB-PAS staining indicated that both ICAld and *L. reuteri* HG001 alleviated CTX-induced intestinal barrier damage. In contrast, the antagonist caused loss of colonic integrity, altered crypt structure, and increased mucosal permeability (Fig. 8L). AHR immunofluorescence revealed that both ICAld and *L. reuteri* HG001 promoted AHR activation in the gut, an effect suppressed by CH-223191 (Fig. S8-1A). Correlation analysis confirmed a positive relationship between AHR expression in tumor tissue and CTX effectiveness (Fig. S8-2D). These *in vivo* results demonstrate that *L. reuteri* HG001 and its metabolite ICAld enhance the chemosensitivity of DLBCL to CTX and reduce CTX-induced intestinal barrier dysfunction and inflammatory responses by activating the AHR, inhibiting the PI3K/AKT/mTOR pathway, and promoting apoptosis.

## Discussion

Although the therapeutic potential of gut microbiota in various malignancies has been widely recognized [21–23], its specific mechanistic role and prognostic value in DLBCL remain poorly understood. This critical knowledge gap has significantly impeded the development of microbiota-targeted therapeutic strategies for DLBCL. To address this limitation, an integrated multiomics approach was used, revealing that gut dysbiosis in patients with DLBCL is characterized by a specific reduction in beneficial bacterial populations rather than an expansion of pathogenic species. Furthermore, this distinctive pattern of microbiota imbalance is accompanied by tryptophan metabolism imbalance (characterized by significantly lower ICAld levels in patients compared with healthy controls) and coexists with disease progression. These findings challenge the traditional “pathogen-centric” view and reveal that the depletion of protective microbiota (predominantly represented by *L. reuteri*), along with the deficiency of its key tryptophan metabolite ICAld, collectively constitute an important microbial mechanism underlying the pathogenesis and progression of DLBCL. Importantly, *L. reuteri* HG001 and its tryptophan metabolite ICAld were identified as key microbial determinants capable of modulating DLBCL progression. The underlying molecular mechanism was fully elucidated: ICAld-mediated activation of the AHR signaling pathway leads to CYP1A1 up-regulation, and the resulting ROS generation synergizes with CTX-induced oxidative stress to collectively suppress the PI3K/AKT/mTOR pathway and promote mitochondrial apoptosis. This mechanistic insight not only provides a novel target for microbiota-based intervention in DLBCL but also establishes a conceptual framework for “microbial metabolite–drug interactions”, thereby laying a solid foundation for future translational research.

In clinical sample validation, significant differences in gut microbiota composition were confirmed between patients with DLBCL and healthy controls. Specifically, beneficial bacterial species, represented by *L. reuteri* HG001, were significantly enriched in healthy individuals but markedly reduced in patients. Subsequently, using culturomics, *L. reuteri* HG001 was identified as a key species that distinguishes patients with DLBCL from healthy individuals. Its relative abundance was significantly lower in patients, and this reduction was negatively correlated with tumor burden, disease severity, clinical stage, and chemotherapy effectiveness. This finding aligns with results from 2 independent cohorts of natural killer/T cell lymphoma, suggesting that the loss of protective microbiota may be a common feature in lymphomagenesis [24,25]. *L. reuteri*, a core human commensal bacterium, has been widely recognized for its probiotic roles across various fields, including metabolic diseases [26,27], neurological disorders [28,29], and tumor immune regulation [18,30]. Previous studies have shown that this bacterium and its metabolite ICAld can improve the effectiveness of immune checkpoint inhibitors in melanoma treatment by promoting the expansion of type 1 cytotoxic T cells, activating the AHR signaling pathway in CD8^+^ T cells, and increasing interferon-γ production [31,32]—a trend largely consistent with the findings in this study.

To further validate the reproducibility of the above findings *in vivo*, a DLBCL-bearing mouse model was established and subjected to the following interventions: FMT from healthy donors, patients with DLBCL, and oral gavage with *L. reuteri* HG001, each combined with chemotherapy. Both healthy donor FMT and *L. reuteri* HG001 supplementation significantly reduced tumor burden and systemic inflammation. Specifically, mice treated with healthy microbiota or *L. reuteri* HG001 showed reduced tumor growth, as indicated by smaller tumor size and weight, a lower proliferation index (decreased Ki67- and p53-positive cells), and increased apoptosis. Concurrently, the expression of intestinal tight junction proteins, including ZO-1 and occludin, was markedly up-regulated, indicating effective mitigation of chemotherapy-induced intestinal barrier damage. These results align with previous studies demonstrating that *L. reuteri* and its metabolites enhance the expression of barrier-related proteins such as ZO-1 and occludin, modulate cytokines associated with intestinal epithelial stemness [33,34], promote crypt stem cell proliferation and epithelial regeneration, and stimulate mucin secretion by goblet cells—collectively reinforcing the chemical barrier function [35]. Furthermore, *L. reuteri* has been shown to activate type 3 innate lymphoid cells to produce IL-22, which, in turn, promotes tight junction protein expression, thereby maintaining intestinal mucosal integrity and immune homeostasis through multiple mechanisms [36,37]. The intervention effects observed in this DLBCL mouse model—specifically, the alleviation of intestinal toxicity and enhancement of chemotherapy response following *L. reuteri* HG001 administration is highly consistent with the mechanisms described above, supporting its potential therapeutic value in the treatment of DLBCL. In addition, other metabolites derived from lactic acid bacteria have been demonstrated to possess immunomodulatory functions. For instance, a novel exopolysaccharide isolated from *Bifidobacterium longum subsp*. *infantis* by Hussein et al. [38] exhibited significant immunomodulatory activity in an lipopolysaccharide-induced inflammatory rat model, stimulating the production of the anti-inflammatory cytokine IL-10 while inhibiting the release of the proinflammatory cytokine IL-6. This further supports the important role of *Lactobacillus* metabolites in regulating host immunity.

Building upon the aforementioned research, through metabolomic screening combined with functional validation *in vitro* and *in vivo*, we identified ICAld as one of the key effector molecules of *L. reuteri* HG001 in its anti-DLBCL activity. *In vitro* experiments demonstrated that ICAld exerted significantly stronger effects than ILA in inhibiting proliferation, inducing apoptosis, and disrupting mitochondrial membrane potential in DLBCL cells. *In vivo* studies further confirmed that ICAld inhibited tumor growth in DLBCL-bearing mice in a dose-dependent manner. Collectively, this evidence suggests that ICAld plays a central role in the anti-DLBCL effect of *L. reuteri* HG001. As a classic endogenous ligand for the AHR, ICAld promotes the formation of the AHR–aryl hydrocarbon receptor nuclear translocator complex upon activation, which binds to xenobiotic response elements and directly regulates the transcription of metabolic enzyme genes such as CYP1A1 and CYP1B1 [39–41]. However, it is important to note that this study did not directly compare the antitumor efficacy of ICAld and ILA *in vivo*, and thus the possibility that ILA may exert synergistic or alternative effects *in vivo* cannot be entirely excluded.

**Fig. 9.**
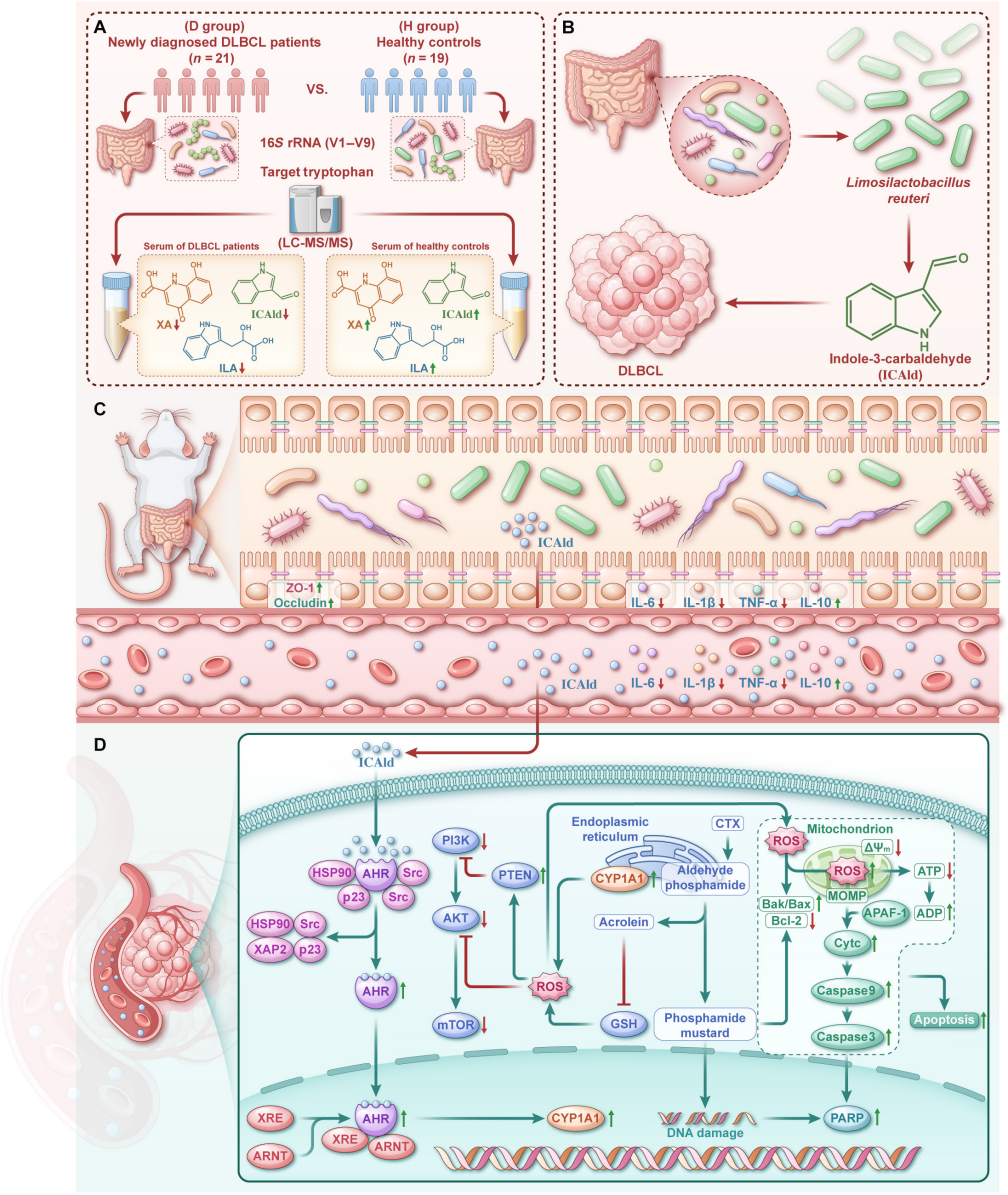
Schematic diagram illustrating the mechanism. (A) Schematic diagram of clinical study. (B) Schematic diagram of metabolomics. (C) Schematic diagram of intestinal changes in animal experiments. (D) Related mechanisms in tumor tissue.This diagram summarizes the mechanism by which *L. reuteri* HG001 and its metabolite indole-3-carbaldehyde (ICAld) alleviate diffuse large B cell lymphoma (DLBCL) progression and enhance chemotherapy efficacy. ICAld activates the aryl hydrocarbon receptor (AHR) signaling pathway, up-regulating cytochrome P450 family 1 subfamily A member 1 (CYP1A1) expression. The resulting reactive oxygen species (ROS) production synergizes with cyclophosphamide (CTX)-induced oxidative stress, collectively inhibiting the phosphatidylinositol 3-kinase (PI3K)/AKT/mechanistic target of rapamycin (mTOR) pathway and promoting mitochondrial apoptosis. GSH, glutathione (reduced form); ATP, adenosine triphosphate; ADP, adenosine diphosphate; HSP90, heat shock protein 90; XAP2, aryl hydrocarbon receptor-associated protein 9; XRE, xenobiotic response element; ARNT, aryl hydrocarbon receptor nuclear translocator; Cytc, cytochrome c; Bak, Bcl-2-antagonist killer; APAF-1, apoptotic protease-activating factor 1; MOMP, mitochondrial outer membrane permeabilization.

The AHR-induced CYP1A1 belongs to the cytochrome P450 family, along with CYP2C9 and CYP3A4, key enzymes responsible for CTX metabolism [42,43]. Hepatic P450 enzymes metabolize CTX into aldophosphamide [44], which subsequently decomposes in tumor cells to form phosphoramide mustard and acrolein: The former induces DNA cross-linking, disrupts DNA structure, and activates the mitochondrial apoptosis pathway, accompanied by substantial ROS generation [45]; the latter further exacerbates ROS accumulation by depleting glutathione and inhibiting the mitochondrial electron transport chain [44,46]. Concurrently, CYP1A1, functioning as a monooxygenase, consumes the reduced form of nicotinamide adenine dinucleotide phosphate and activates molecular oxygen during the catalysis of substrates such as ICAld, leading to the production of oxygen radical intermediates that also contribute significantly to ROS generation [47,48]. With increasing ICAld concentrations, AHR is persistently activated, further up-regulating CYP1A1 expression and forming a positive feedback loop characterized by dysregulated oxidative stress [49]. ROS generated via CYP1A1 metabolism synergizes with that produced during CTX metabolism, collectively enhancing tumor cell apoptosis induction and suppressing PI3K/AKT/mTOR signaling pathway activation [50]. In tumor tissues from ICAld-treated DLBCL-bearing mice, elevated expression of the proapoptotic proteins Bax, cleaved-caspase3, and p-PARP, together with enhanced AHR expression, was observed. Conversely, phosphorylation of key proteins in the PI3K/AKT/mTOR pathway was inhibited, while expression of its negative regulator PTEN was significantly up-regulated. These results provide a comprehensive molecular-level explanation of the mechanism by which *L. reuteri* HG001 and its metabolite ICAld enhance the antitumor efficacy of CTX and ameliorate CTX-induced intestinal barrier injury. (A detailed schematic of the proposed mechanism is provided in Fig. 9.) In contrast to the present study, which focuses on the mechanism by which ICAld regulates tumor cell apoptosis through the AHR pathway, the research by Luo et al. [51] has revealed another crucial role of ICAld in an atherosclerosis model—namely, its ability to promote macrophage cholesterol efflux and suppress inflammatory responses by up-regulating miR-1271-5p to inhibit histone deacetylase 9 expression. This finding suggests that the biological functions of ICAld are pleiotropic, and while its mechanisms of action vary across different disease models depending on cell type and microenvironmental context, they collectively underscore its central role as a key immunomodulatory molecule.

Notably, although the mechanism by which ICAld induces ROS production via the AHR/CYP1A1 pathway is logically coherent, its potential off-target effects—specifically, the risk of oxidative damage to normal cells—represent a critical consideration in evaluating the clinical translatability of this strategy. To address this concern, we compared the differential effects of ICAld on tumor cells (SU-DHL-4 and HT29) versus their normal counterparts (GM12878 and NCM460). The results demonstrated that, at the same intervention concentration, ICAld significantly elevated ROS levels and inhibited cell viability only in tumor cells, while exerting minimal effects on normal cells. These findings indicate that ICAld possesses clear tumor-selective cytotoxicity, a phenomenon likely attributable to the “oxidative stress vulnerability” of cancer cells: Although tumor cells typically exhibit elevated basal ROS levels and compensatory up-regulation of antioxidant systems, they are paradoxically more susceptible to additional oxidative insults [52,53]. In contrast, normal cells harbor sufficient antioxidant reserves to buffer equivalent ROS challenges. Thus, the ICAld-induced ROS production via the AHR/CYP1A1 pathway may selectively exceed the antioxidant compensatory threshold of tumor cells, triggering apoptosis without causing significant damage to normal cells [54]. This selectivity not only provides critical biological plausibility for the mechanistic hypothesis proposed in this study but also preliminarily confirms that the ICAld-combined chemotherapy strategy possesses an acceptable therapeutic window, thereby laying a foundation for its subsequent clinical translation.

In summary, this study has first demonstrated that the main feature of gut dysbiosis in patients with DLBCL is a widespread decrease in beneficial bacteria, with *L. reuteri* HG001 and its metabolite ICAld playing a key role in disease progression and response to chemotherapy. ICAld was shown to regulate oxidative stress, cause mitochondrial damage and apoptosis, enhance the effects of CTX, and repair chemotherapy-induced intestinal barrier damage through AHR activation. This finding opens new possibilities for microbiota-based approaches to preventing and treating DLBCL.

Despite these innovative findings, several limitations of this study must be acknowledged. First, the clinical sample size was relatively small; future validation of the reliability of these microbial markers through multicenter, large-scale cohorts is necessary. Second, the full-length 16*S* sequencing used in the human cohort provides only relative abundance data, highlighting the need for target-specific quantitative polymerase chain reaction (qPCR) to enable absolute quantification of key bacteria and more accurately assess their association with clinical indicators. Moreover, it remains unclear whether other metabolic pathways of *L. reuteri* HG001, such as the kynurenine pathway, contribute to its antitumor effects. Furthermore, beyond the PI3K/AKT/mTOR pathway, it is worth exploring whether ICAld influences DLBCL progression by modulating epigenetic regulation or other signaling networks. Most importantly, the safety and efficacy of *L. reuteri* HG001 or ICAld have not yet been validated in humans, which is a crucial step toward clinical application. However, it is important to note that the present study did not directly compare the antitumor efficacy of ICAld and ILA *in vivo*; therefore, the possibility that ILA may exert synergistic or alternative effects *in vivo* cannot be entirely excluded. In addition, although we used a standard antibiotic depletion protocol for FMT experiments as reported in the literature, we did not perform 16*S* sequencing or qPCR validation on fecal samples from mice before and after antibiotic treatment, thus lacking direct molecular evidence for the efficacy of endogenous microbiota depletion.

## Materials and Methods

### Human studies

A clinical cohort of 40 participants was established through recruitment at the Second Affiliated Hospital of Nanchang University. The DLBCL group (*n* = 21) included newly diagnosed patients who met the diagnostic criteria of the Chinese Society of Clinical Oncology Lymphoma Guidelines (2024 revision) for DLBCL and had not received any antibiotic or probiotic therapy recently. Pathological subtypes were categorized into 2 groups, namely, GCB and activated B-cell-like. A control group (*n* = 19) was matched to the DLBCL participants by age and sex. Exclusion criteria were (a) relapsed or refractory DLBCL; (b) presence of gastrointestinal disorders or systemic diseases (e.g., severe organ dysfunction, active infections, rheumatic diseases, or other malignancies); (c) use of antibiotics, immunosuppressants, or probiotics within the preceding 6 months or long-term use of medications not indicated for DLBCL management per guidelines; (d) pregnancy; and (e) inability or unwillingness to provide written informed consent. Upon collection, fecal samples were promptly mixed with 30% glycerol for stabilization and were placed at −80 °C for long-term storage within 2 h.

The study used full-length 16*S* rRNA gene sequencing (V1 to V9 regions) to analyze the gut microbiota composition. Following DNA extraction from fecal samples, full-length amplicon sequencing was performed to obtain the complete 16*S* rRNA gene sequence [55]. The sequencing data were quality-controlled and aligned against the National Center for Biotechnology Information (https://www.ncbi.nlm.nih.gov/) database to achieve accurate species-level annotation. After identifying differential microbiota through full-length 16*S* sequencing, a culturomics strategy was further adopted to isolate and culture the target strain from fecal samples of healthy volunteers. The isolated strain was subjected to bidirectional Sanger sequencing of the 16*S* rRNA gene, and the resulting sequences were assembled to obtain the full-length sequence for species identification.

Given that this study was a cross-sectional observational study without any interventions, clinical trial registration was not applicable. The study protocol was reviewed and approved by the Ethics Committee of the Second Affiliated Hospital of Nanchang University (approval no. 2025011).

### Animal experiments

All experiments were conducted with mice housed in a designated pathogen-free facility under a 12-h light–dark cycle. The animals, healthy female NSG mice aged 4 to 6 weeks, were allowed free access to standard food and water. All NSG mice were obtained from Shanghai Model Organism Center. After 1 week of acclimatization, (a) experiment 1 involved the subcutaneous inoculation of DLBCL cells (SU-DHL-4, 5 × 10^6^ cells/100 μl) to establish xenograft models, followed by 1 week of antibiotic treatment to deplete the gut microbiota. FMT was initiated from week 2, with intraperitoneal chemotherapy (CTX, 30 mg/kg, administered biweekly over the course of 3 weeks) between weeks 6 and 8. The study evaluated the impact of FMT on DLBCL progression in MCH and MCD groups, with continuous monitoring of body weight and tumor volume. At the end of the study, fecal samples were collected for analysis. Blood samples were obtained immediately following euthanasia. To prepare serum, the blood was allowed to clot at room temperature for 2 h and then centrifuged at 3,000 rpm for 15 min at 4 °C; the resulting supernatant (serum) was collected for subsequent assays. Tumors and colons were isolated, photographed, and fixed in 4% paraformaldehyde for histopathological/immunohistochemical evaluation or stored at −80 °C for molecular studies. (b) Experiment 2 isolated *L. reuteri* HG001 to investigate its functional effects on DLBCL. The strain was cultured in MRS broth for 24 to 36 h, and mice received 100 μl of bacterial suspension via oral gavage every other day (the concentration of the *L. reuteri* HG001 suspension was adjusted to 1 × 10^9^ colony-forming units (CFU)/ml; each mouse received a daily oral gavage of 0.1 ml of this suspension, corresponding to a daily dose of 1 × 10^8^ CFU per mouse; the control group received an equal volume of gelatin-normal saline via gavage). Chemotherapy (CTX) was initiated intraperitoneally at week 5, with data collection and sample processing identical to experiment 1. (c) Experiment 3 examined the role of tryptophan metabolism in *L. reuteri*-mediated CTX efficacy. The MCL_neg group was fed tryptophan-deficient diets, while the controls received standard chow; subsequent procedures were identical to those of experiment 1. (d) Experiment 4 evaluated the DLBCL-modulating effects of *L. reuteri*-derived tryptophan metabolite ICAld, administered via drinking water at graded concentrations (50, 100, and 200 mM) with CTX initiation at week 5. Collected tissues (tumors, colon, heart, liver, spleen, kidneys, and lungs) underwent fixation or cryopreservation. At the same time, blood samples were processed for hematological and biochemical analyses as in experiment 1. (e) Experiment 5 investigated whether ICAld synergized with CTX by activating the AHR. Cohorts received intraperitoneal AHR antagonist CH-223191 (5 mg/kg, twice every other day) before CTX, with protocols otherwise consistent with prior experiments. The Animal Welfare and Ethics Committee at Nanchang University approved all experiments involving animals (approval no. NCULAE-20241115001).

The *L. reuteri* strain used in this study was isolated from fecal samples of healthy adult donors. It has been deposited in the China General Microbiological Culture Collection Center (CGMCC no. 36248) under the designation *L. reuteri* HG001. The complete genome sequence of this strain and the certificate of deposit is provided in the Supplementary Materials.

### Cell experiments

All 4 cell lines were cultured under standard conditions (37 °C and 5% CO_2_). Specifically, SU-DHL-4 and GM12878 cells were maintained in RPMI 1640 complete medium supplemented with 10% and 15% fetal bovine serum (FBS), respectively; OCI-LY3 cells were cultured in Iscove’s modified Dulbecco’s medium containing 20% FBS; and NCM460 cells were cultured in RPMI 1640 medium supplemented with 10% FBS. For cell lines other than NCM460, the culture media were additionally supplemented with penicillin (100 U/ml) and streptomycin (100 μg/ml) to reduce the risk of contamination during culture. NCM460 normal colon epithelial cells were cultured in the absence of antibiotics to avoid potential effects on cellular physiological functions. The SU-DHL-4 and GM12878 cell lines were obtained from the Cell Bank of the Chinese Academy of Sciences, while the OCI-LY3 and NCM460 cell lines were purchased from iCell Bioscience Inc. (Shanghai).

1. Experiment 1: Investigation of functional components in *L. reuteri* HG001 after 20-h coincubation with CTX. Cells were then further treated with different bacterial components (heat-inactivated *L. reuteri* HG001, bacterial supernatant, or live bacteria) for 4 h. Experimental groups included control (C: CTX + MRS medium), CTX + *L. reuteri* HG001 (CL), CTX + supernatant (CS), and CTX + heat-inactivated *L. reuteri* HG001 (CH). Negative controls assessed the tumor-suppressive effects of bacterial components in the absence of CTX.
2. Experiment 2: Identification of active metabolites in supernatant based on targeted tryptophan metabolomics, 3 metabolites (ICAld, ILA, and XA) were compared for efficacy. Cells were preincubated with metabolites for 12 h before CTX addition for another 12 h (total 24 h). Groups were control (C: CTX + phosphate-buffered saline [PBS]), CTX + ICAld (0.5 mM; CI), CTX + ILA (0.5 mM; CL), and CTX + XA (0.5 mM; CA). Negative controls evaluated metabolites alone.
3. Experiment 3: To evaluate whether ICAld exerts selective cytotoxicity toward tumor cells, this study selected the human DLBCL cell line SU-DHL-4, the normal human B lymphocyte cell line GM12878, the human colorectal adenocarcinoma cell line HT29, and the normal human colon epithelial cell line NCM460. A control group (vehicle) and a treatment group (ICAld; 0.5 mM) were established for each cell line, with a treatment duration of 12 h. The groups were set as follows: control (vehicle) and ICAld (0.5 mM). ROS levels were assessed using flow cytometry (*n* = 3), and cell proliferation was evaluated using the CCK-8 assay (*n* = 5).
4. Experiment 4: AHR antagonist study—the AHR-mediated effect of ICAld was investigated using the AHR antagonist CH-223191. After 12 h of coincubation with ICAld and CH-223191, CTX was added for an additional 12 h (totaling 24 h). Groups included control (C: CTX + PBS), CTX + CH-223191 (10 μM; CI), CTX + ICAld (0.5 mM; CA), and CTX + ICAld + CH-223191 (CAI). Negative controls examined ICAld’s AHR-dependent effects in the absence of CTX.

### Microbiome analysis

Total DNA was extracted from −80 °C preserved fecal samples using the PersonalBio Genomic DNA Extraction Kit (Shanghai, China), following the manufacturer’s protocol. The V1 to V9 regions of the bacterial 16*S* rRNA gene were then amplified with universal primers. The resulting amplicons were purified and sequenced on an Illumina NovaSeq 6000 platform (paired-end). Raw sequencing reads were processed with Vsearch (v2.13.4) and cutadapt (v2.3) to remove adapter/primer sequences and low-quality bases, generating high-quality amplicon sequence variants or operational taxonomic units. The length distribution of these final sequences was also assessed. Taxonomic annotation was performed using the classify-sklearn algorithm in QIIME2, where amplicon sequence variant/operational taxonomic unit sequences were classified against reference databases (Greengenes, NT, UNITE) via pretrained naive Bayes classifiers across 7 taxonomic ranks (domain to species). Subsequent analyses encompassed community composition assessment, α- and β-diversity analyses, differential species identification, biomarker detection, and co-occurrence network construction.

### Metabolomic analysis

Using LC-MS/MS, we performed targeted metabolomics to measure 22 tryptophan metabolites in fecal and human serum specimens. Subsequent data analysis relied on a custom-built metabolome database (MWDB, Majorbio Bio-Pharm Technology, Wuhan, China). Quantitative MS data were acquired in multiple reaction monitoring mode using a QTRAP 6500+ triple quadrupole mass spectrometer (SCIEX, MA, USA) and processed with MultiQuant 3.0.3 software (SCIEX). Peak integration and correction were performed based on reference standard data. Subsequently, metabolite concentrations were determined by converting peak area ratios via linear calibration curves. Finally, multivariate statistical analyses—including principal components analysis, orthogonal projections to latent structures discriminant analysis, and clustering—were applied to the processed metabolomic data to screen for differential metabolites and uncover key changes in metabolic profiles.

### Fecal microbiota transplantation

Following a 1-week antibiotic pretreatment (vancomycin [100 mg/kg per day], neomycin [200 mg/kg per day], metronidazole [200 mg/kg per day], and ampicillin [200 mg/kg per day]) to clear gut flora, mice received FMT. Donor feces from eligible patients with DLBCL and healthy controls were pooled separately by group, homogenized in saline, filtered through a 0.45-μm filter, and centrifuged (100*g* for 5 min at 4 °C); the supernatant was cryopreserved in 30% glycerol [56]. For administration, aliquots were thawed, centrifuged, resuspended in gelatin-saline, and gavaged.

### *In Vitro* growth, tolerance, and antimicrobial activity of *L. reuteri* HG001

Overnight culture of the *L. reuteri* HG001 strain was carried out in MRS broth after an initial activation period. Optical density (OD) at 600 nm was measured every 2 h until OD plateaued (indicating the stationary phase), generating growth curves with time as the independent variable and OD as the dependent variable. For bile salt and acid tolerance testing [57], *L. reuteri* HG001 was grown in MRS broth supplemented with bovine bile salts (0% to 0.3%) or acidified to pH 3 to 6; viable counts were determined from stationary-phase cultures. Antimicrobial activity was tested by applying *L. reuteri* HG001 culture supernatant (250 μl in Oxford cups) to lawns of indicator pathogens on LB agar and measuring inhibition zones after 8 h. Using the Kirby–Bauer disk diffusion method (CLSI 2018) [58], antibiotic susceptibility was assessed by applying disks to agar plates seeded with bacterial suspensions and measuring inhibition zones at 24 h.

### Hematological analysis

For hematological profiling, parameters (red blood cell, white blood cell with differential, and platelet) were measured from blood in glycerol tubes using a Mindray BC-5000 Vet analyzer. For serum biochemistry, samples stored at −80 °C were analyzed on a Beckman Coulter AU480 system to determine levels of alanine aminotransferase, aspartate aminotransferase, and creatine kinase-MB. In addition, the concentrations of inflammatory cytokines (TNF-α, IL-6, IL-1β, and IL-10) in serum were determined with commercial enzyme-linked immunosorbent assay kits from Wuhan Boster Biotechnology, strictly adhering to the manufacturer’s guidelines. In addition, target analytes (ICAld) in serum, colon, and tumor tissue samples, as well as tumor-related hematological indicators (SF, LDH, and β2-MG), were measured using commercially available kits.

### The evaluation of DLBCL progression

Upon complete resection of tumor tissue, specimens were initially rinsed with sterile physiological saline or PBS to remove residual fixatives. Following precise measurement of tissue weight and volume, portions of the specimens were immediately immersed in 4% neutral-buffered formaldehyde for fixation. In contrast, the remaining tissues were rapidly transferred to precooled liquid nitrogen for snap-freezing, designated for subsequent qPCR quantification and Western blot protein analysis. Formaldehyde-fixed tumor tissues underwent graded dehydration in 25% sucrose/PBS solution for 48 h (sinking to the bottom indicating completion of dehydration). After embedding in optimal cutting temperature compound (cryomolds) and cryostat sectioning, the tissue blocks were foil-wrapped, labeled, and stored at −80 °C in preparation for downstream histopathological and molecular analyses. For staining analysis, routine HE staining was combined with immunohistochemical staining (including markers such as Ki67 and p53) to evaluate tumor proliferative activity and mutational status. All stained sections underwent digital quantitative analysis using ImageJ (developed by the National Institutes of Health), ensuring objectivity and reproducibility in data acquisition.

### Histology and immunohistochemistry analysis

In the histopathological detection system, comprehensive analytical methods for tumor tissue and multiorgan specimens (including colon, heart, liver, spleen, lung, and kidney) are systematically implemented as follows: Initially, routine HE staining and morphological evaluation serve as the foundation for pathological analysis [59]. The standardized protocol involves preparing 4-μm-thick sections from paraffin-embedded tissue samples, followed by HE staining and microscopic examination under a Nikon Eclipse 80i optical microscope, with subsequent digital assessment of structural lesions and morphological features utilizing NIS-Elements 3.2 specialized image analysis software. For specialized colon tissue analysis, the AB-PAS special staining technique is used to quantify goblet cells, combined with measurement of crypt depth, thereby comprehensively evaluating the integrity of the intestinal mucosal barrier function [60]. In the immunohistochemical detection phase [61], strict adherence to standardized protocols is maintained. Initial antigen retrieval is performed using citrate buffer (pH 6.0), followed by a 1-h incubation with a bovine serum albumin blocking solution to prevent nonspecific binding. Sequential antigen–antibody reactions include primary antibody incubation (overnight at 4 °C) and horseradish-peroxidase-labeled secondary antibody incubation (50 min at 37 °C). Final sample preparation includes hematoxylin counterstaining, gradient dehydration, and neutral balsam mounting, with all stained sections evaluated for positive expression under Nikon microscopy. For samples requiring higher-resolution analysis, dual-label immunofluorescence techniques are used. Frozen sections are permeabilized for 20 min with 0.5% Triton X-100 to enhance membrane permeability, followed by a 1-h blocking step with 3% bovine serum albumin. Subsequent immunofluorescence reactions include primary antibody incubation (overnight at 4 °C) and incubation with fluorescein isothiocyanate (FITC)- or cyanine3-labeled secondary antibodies (2 h in the dark). Final processing involves fixation with antifluorescence quenching mounting medium, glycerol mounting, and multichannel signal acquisition via fluorescence microscopy.

### RNA sequencing and transcriptomic analysis

In this study, a comprehensive gene expression analysis system was established using the SU-DHL-4 cell line. The experimental design used a control–treatment comparison model, with the control group (group C) consisting of untreated DLBCL cells (*n* = 5) and the experimental group (group I) comprising ICAld-treated DLBCL cells (*n* = 5). The technical sequencing work (RNA extraction, library prep, and Illumina sequencing) was performed by Personalbio Biotechnology Co. Ltd. (Shanghai). Differentially expressed genes were identified using a dual threshold of *P* < 0.05 and a fold change of ≥1.5. The analysis tools were selected on the basis of the experimental design: DESeq2 for data with biological replicates and edgeR for those without. Data visualization was achieved using heatmaps to depict global patterns of gene expression. During the functional annotation process, GO and KEGG pathway enrichment analyses were performed using algorithms based on the hypergeometric distribution to systematically clarify the biological functional traits and metabolic pathways associated with genes with varying expression levels.

### Western blotting

Protein extracts from cancerous tissues and cell lines (SU-DHL-4/OCI-LY3) were prepared with radioimmunoprecipitation assay lysis buffer (containing protease/phosphatase inhibitors) and quantified by bicinchoninic acid. Equal amounts were separated on 8% to 12% sodium dodecyl sulfate–polyacrylamide gel electrophoresis gels, transferred to polyvinylidene difluoride membranes, and immunoblotted following standard protocols [62]: blocking in 5% skim milk, incubation with specific primary antibodies (overnight, 4 °C), and corresponding horseradish-peroxidase-conjugated secondary antibodies (2 h, room temperature), with tris-buffered saline with Tween 20 (TBST) washes between steps. Finally, 1-min incubation with super ECL chemiluminescent substrates for signal detection using Tanon 5200 automated gel imaging system (Shanghai, China). Data analysis was performed in ImageJ to quantify protein band gray values, ensuring experimental accuracy and reproducibility [63]. Table S3 lists the antibodies used for Western blotting in this study.

### Apoptosis detection

Mitochondrial membrane potential was evaluated with the JC-1 assay kit (Beyotime). Cells (SU-DHL-4, 1.0 × 10^5^ per well; OCI-LY3, 1.5 × 10^5^ per well) seeded in 6-well plates were stained, imaged on a Leica DM i8 fluorescence microscope, and analyzed in ImageJ by calculating the ratio of aggregate (red) to monomer (green) fluorescence intensity. For annexin V/propidium iodide (PI) double staining, the Annexin V–FITC/PI Apoptosis Detection Kit (Elabscience) was used to stain the treated SU-DHL-4 and OCI-LY3 cells. The cells were resuspended to a single-cell suspension (500 μl) and analyzed by flow cytometry (IDH7000, Sony Corporation, Japan), with data processed using FlowJo V.10.9.0.

### Statistical analysis

Statistical details are as follows: Sample sizes (*n*) are provided in figure legends; analyses utilized GraphPad Prism 9, SPLS V2.0, and SPSS Statistics 26. Clinical data, presented as means ± SEM/SD (normal), median (interquartile range) (nonnormal), or frequency (%) (categorical) based on Shapiro–Wilk normality testing, were compared using independent *t* tests, Mann–Whitney *U* tests, or chi-square tests, as appropriate. Spearman correlation (R corrplot) assessed clinical-microbiota relationships. Significance (*P* < 0.05, 2-sided) was determined by one-way analysis of variance (ANOVA) (Dunnett’s) for multiple groups or unpaired *t* tests for 2-group comparisons.

## Ethical Approval

The clinical study protocol was reviewed and approved by the Ethics Committee of the Second Affiliated Hospital of Nanchang University (approval no. 2025011). The Animal Welfare and Ethics Committee at Nanchang University approved all experiments involving animals (approval no. NCULAE-20241115001).

## Data Availability

All sequencing data from this study (including human and mouse 16*S* rRNA data) are publicly available; the raw reads can be retrieved from the National Center for Biotechnology Information Sequence Read Archive under the accession numbers PRJNA1392177, PRJNA1392205, and PRJNA1392348. Further information and requests for resources and reagents should be directed to the lead contact J.L. (ndefy03048@ncu.edu.cn).
