# Supplementary material for: Indole-3-Carbaldehyde from Limosilactobacillus reuteri Boosts Chemotherapy Response in Diffuse Large B Cell Lymphoma by Blocking the Mechanistic Target of Rapamycin Pathway
Source: Research (Wash D C). 2026 May 4;9:1267. doi: 10.34133/research.1267 (PMC13136617; doi:10.34133/research.1267)
Supplement: Supplementary 1 — Figs. S1 to S8 Tables S1 to S4 [file research.1267.f1.pdf]

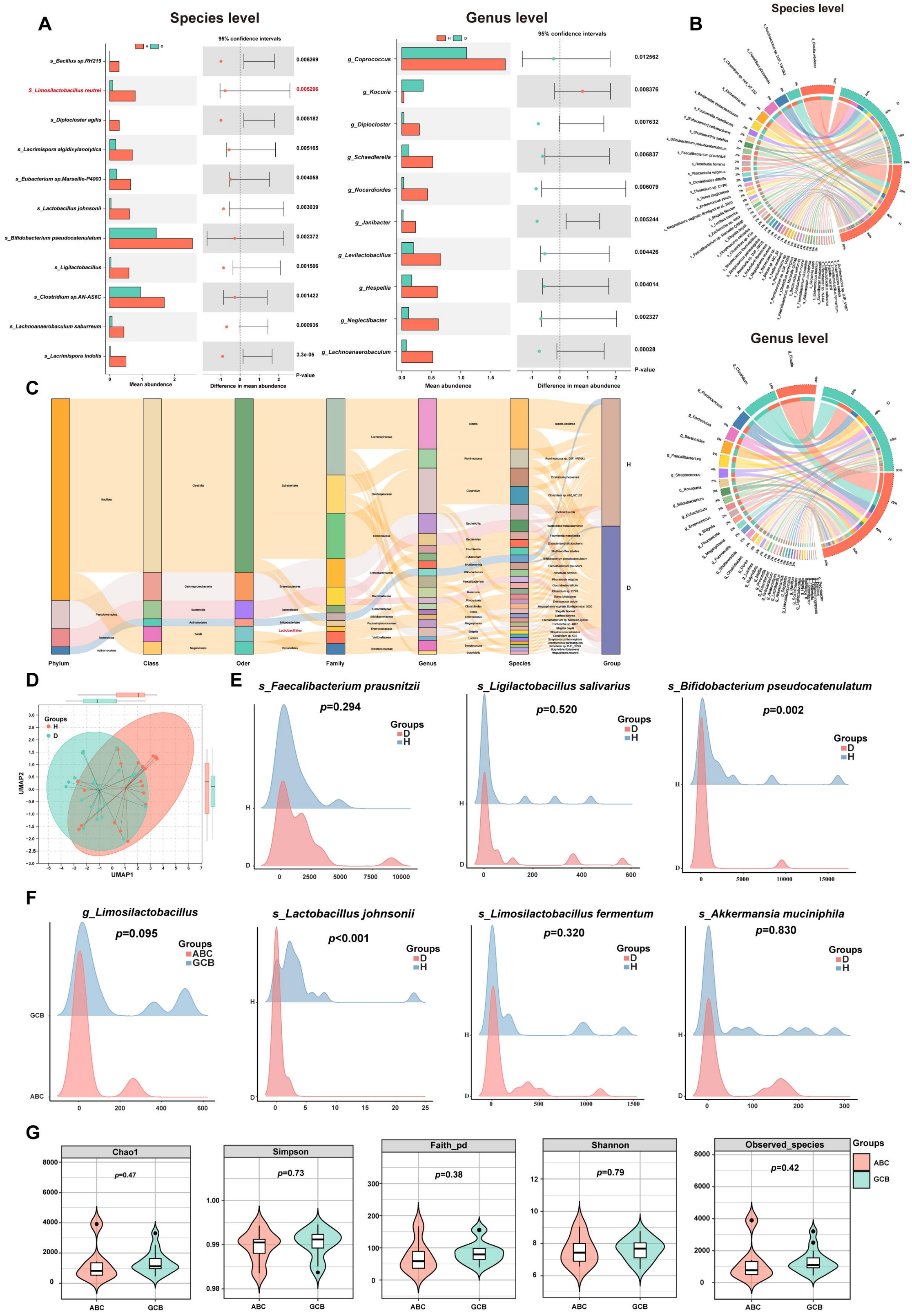

Supplementary Figure 1

(A) Stamp plot of species composition differences at the species and genus level.

(B) Chord diagram of species composition differences at the species and genus level.

(C) Sankey diagram of species composition from the phylum level to the species level.

(D) TSNE/UMAP analysis of beta diversity.

(E) Ridge plots of the abundance of *Limosilactobacillus fermentum*, *Ligilactobacillus salivarius*, *Lactobacillus johnsonii*, *Akkermansia muciniphila*, *Faecalibacterium prausnitzii*, and *Bifidobacterium pseudocatenulatum* at the species level (n = 40).
(F) Ridge plot of the abundance of the genus *Lactobacillus* in two DLBCL subtypes (GCB and ABC) (n = 21).
(G) Alpha diversity of microbial communities in populations with two DLBCL subtypes (GCB and ABC).
Data are presented as mean ± SD. Statistical significance was determined by one-way ANOVA with Dunnett's multiple comparisons test or Mann-Whitney U test. ns, not significant. Spearman correlation analysis was employed to examine the interrelationships among variables. ns, not significant, *$p < 0.05$, **$p < 0.01$, ***$p < 0.001$, ****$p < 0.0001$.

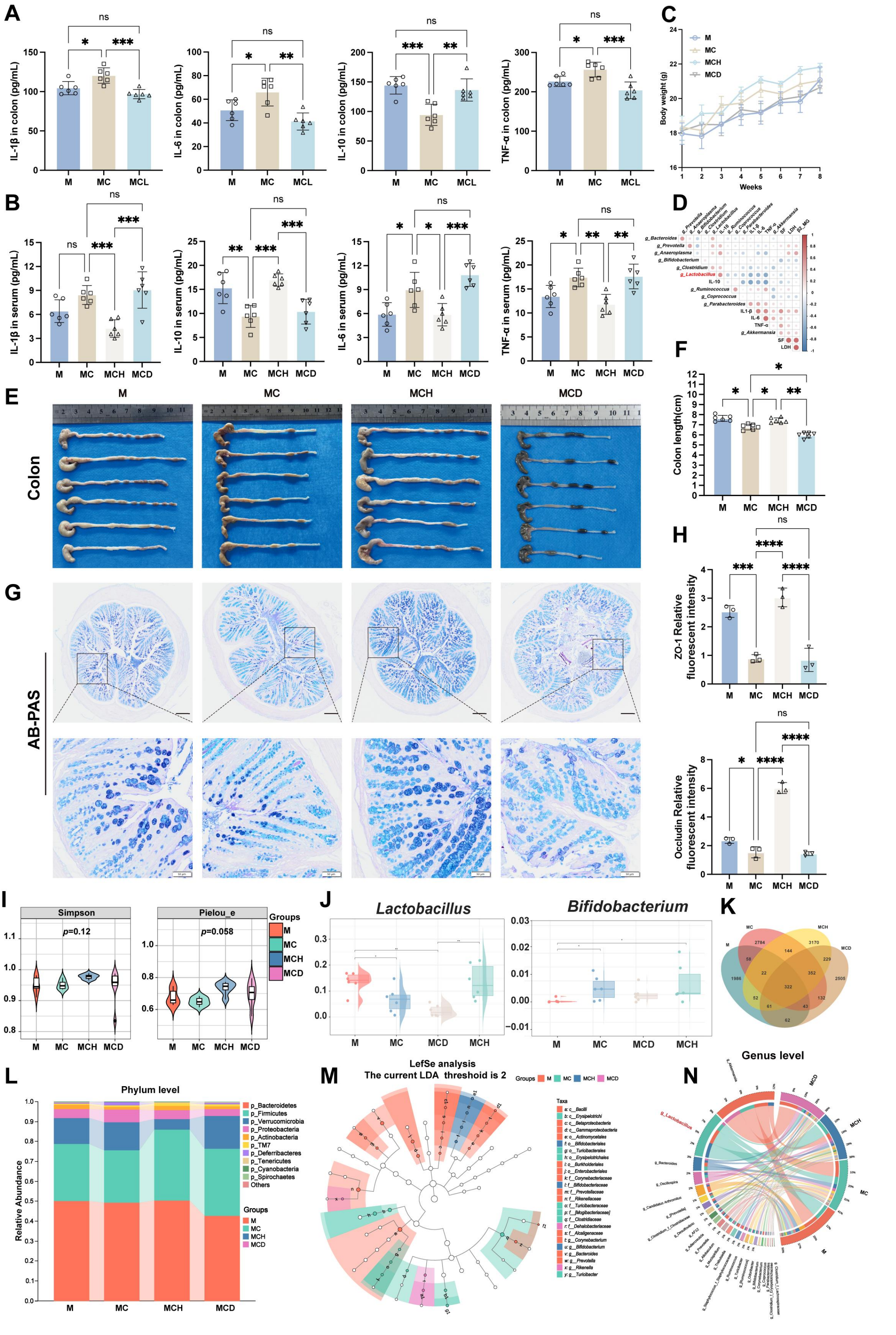

Supplementary Figure 2

(A, B) Levels of inflammatory factors (IL-1β, IL-6, IL-10, TNF-α) in the colon and serum (n = 6).

(C) Curve of body weight changes in mice.

(D) Correlation analysis between inflammatory factors (IL-1β, IL-6, IL-10, TNF-α), blood

indicators related to DLBCL tumor burden (β2-MG, SF, and LDH), and intestinal microbial abundance.
(E) Display of mouse colons from different groups (n = 6).
(F) Bar chart of colon length (n = 6).
(G) Representative images of colon AB-PAS staining ((Scale bars correspond to 50 μm (top) and 20 μm (bottom))).
(H) Bar chart of quantitative analysis of ZO-1 and Occludin immunofluorescence staining in the colon (n = 3).
(I) Alpha-diversity of microbial communities.
(J) Cloud-rain plot of the relative abundance of *Lactobacillus* and *Bifidobacterium*.
(K) Venn diagram of microbial community differences.
(L) Histogram of species composition at the phylum level.
(M) Taxonomic branching diagram of bacterial relative abundance generated based on the linear discriminant analysis (LDA) effect size (LDA > 2) of 16S rRNA gene sequences.
(N) Chord diagram of species composition.
Data are presented as mean ± SD. Statistical significance was determined by one-way ANOVA with Dunnett's multiple comparisons test or Mann-Whitney U test. ns, not significant, $^{*}p < 0.05$, $^{**}p < 0.01$, $^{***}p < 0.001$, $^{****}p < 0.0001$.

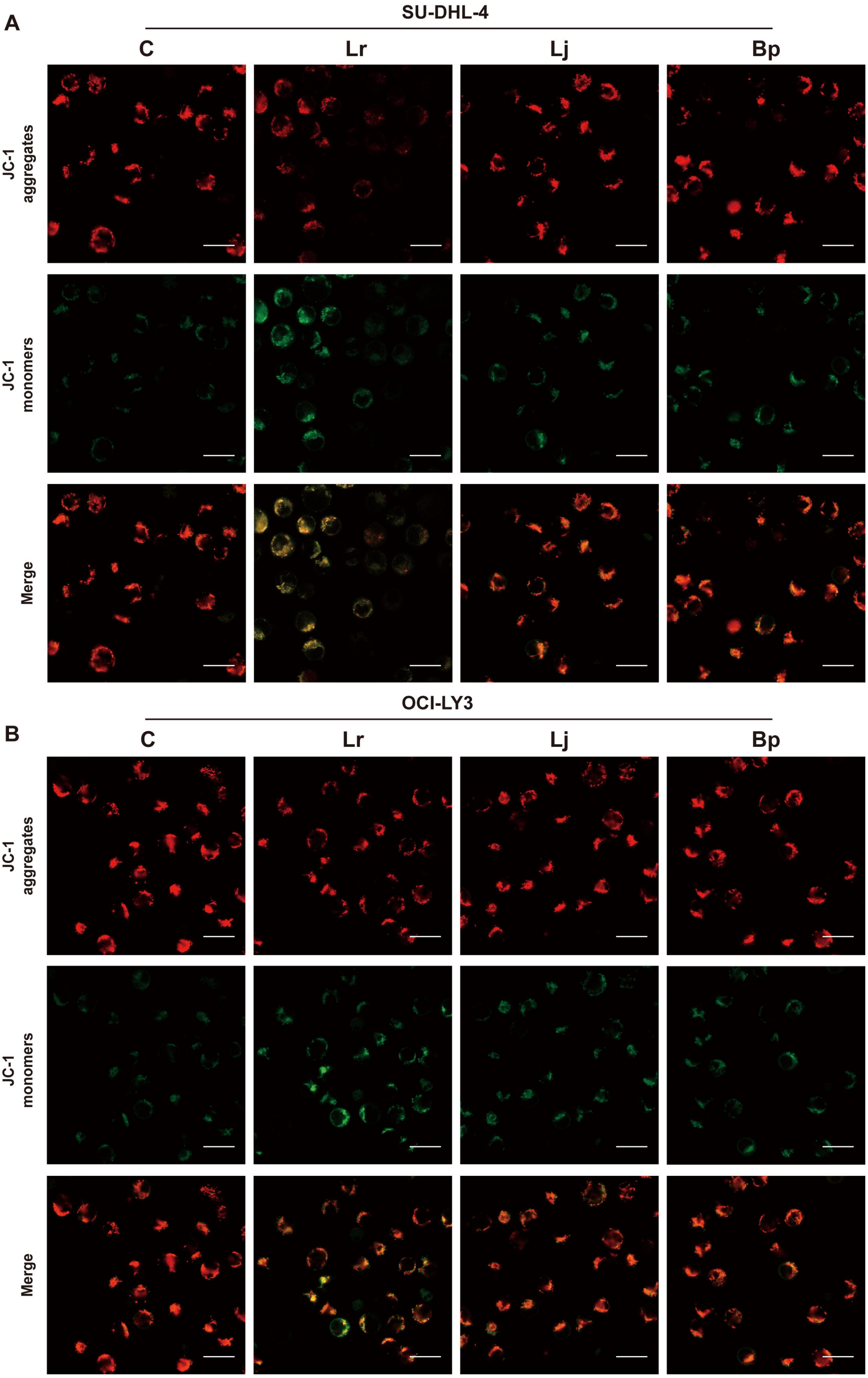

Supplementary Figure 3-1

(A, B) Comparison of mitochondrial depolarization capacity in DLBCL cell lines (SU-DHL-4 and OCI-LY3) induced by three probiotic strains (*Limosilactobacillus reuteri* (Group Lr), *Lactobacillus johnsonii* (Group Lj), and *Bifidobacterium pseudolongum* (Group Bp)), (scale bar = 20 μm).

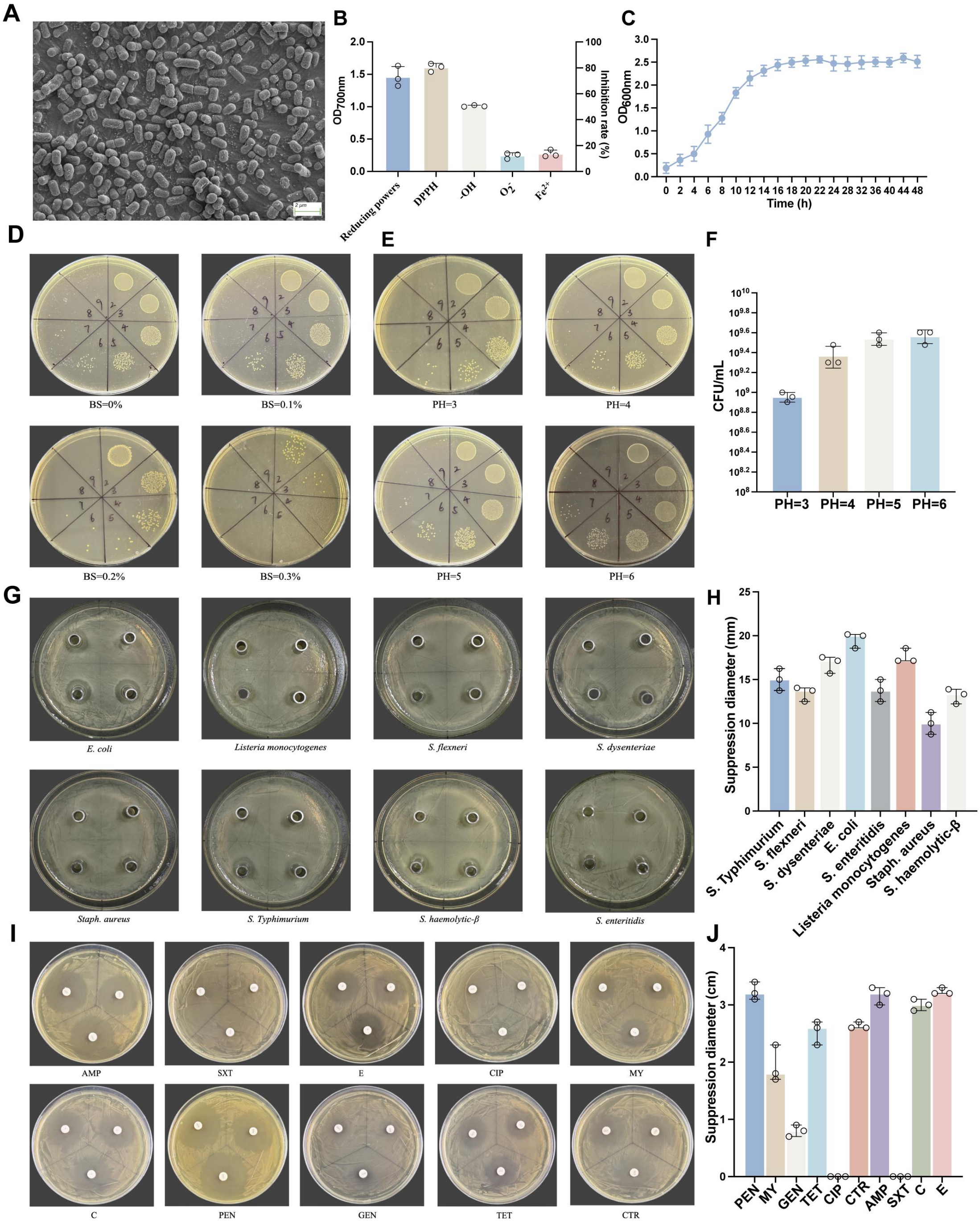

Supplementary Figure 3-2 Identification of probiotic properties of *L.reuteri* HG001.

(A) Electron microscopy image (scale bar = 2 μm).

(B) Assessment of antioxidant properties.

(C) Growth curve analysis.

(D) Schematic representation of bile salt tolerance experiments.

(E) Schematic representation of acid tolerance experiments.

(F) Bar chart depicting the diameters of inhibition zones in acid tolerance experiments (n = 3).

(G) Schematic representation of antibacterial experiments.

(H) Bar chart depicting the diameters of inhibition zones in antibacterial experiments (n = 3).

(I) Antibiotic susceptibility testing via the disk diffusion method; AMP, ampicillin (10 μg / tablet); SXT, Compound sulfamethoxazole (25 μg / tablet); E, erythromycin (15 μg / tablet); CIP, ciprofloxacin (5 μg / tablet); MY, lincomycin (2 μg / tablet); C, chloramphenicol (30 μg / tablet); PEN, penicillin (10 μg / tablet); GEN, gentamycin (10 μg / tablet); TET, tetracycline (30 μg / tablet); CTR, ceftriaxone (30 μg / tablet).

(J) Bar chart depicting the diameters of inhibition zones in antibiotic susceptibility testing (n = 3).

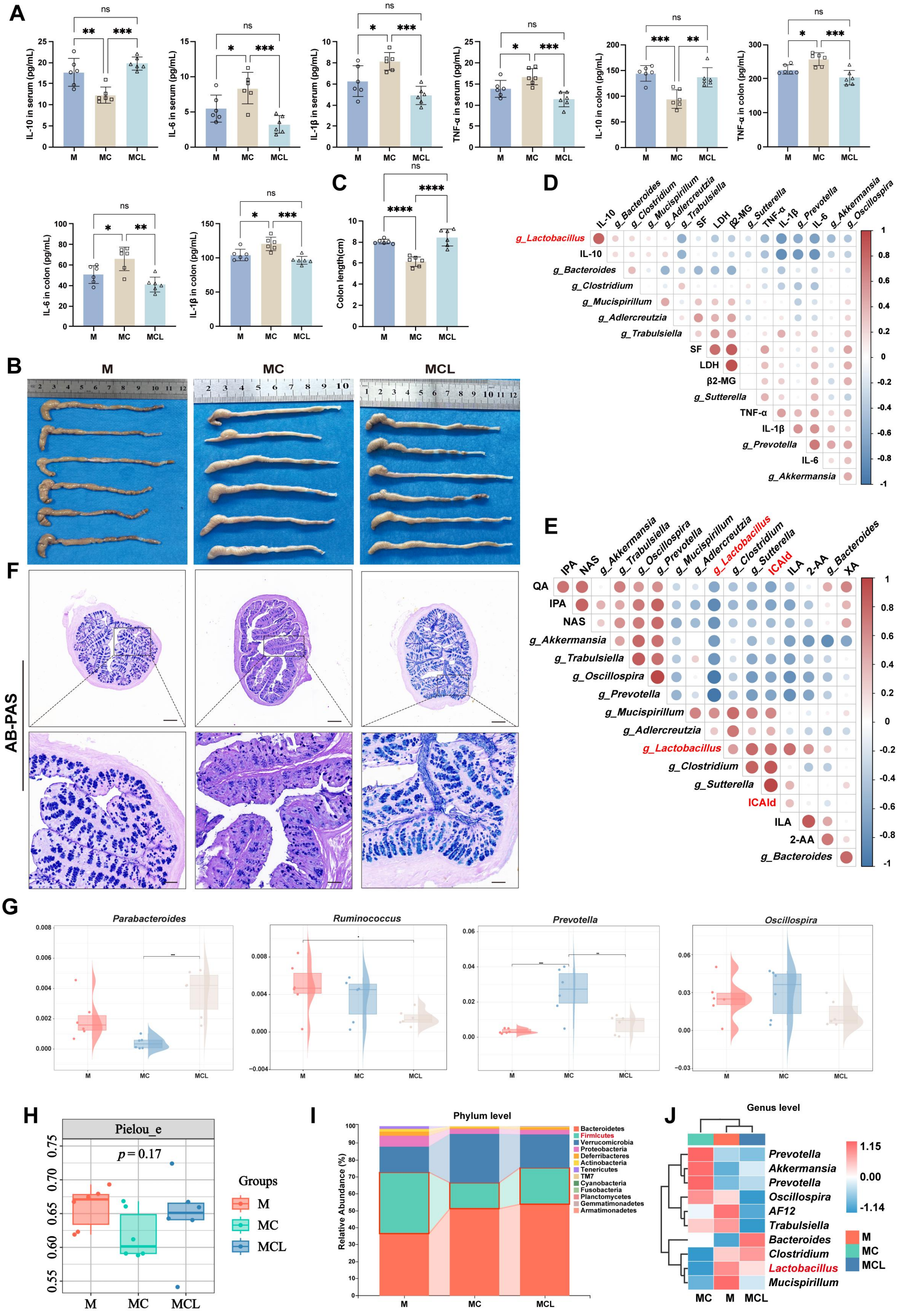

Supplementary Figure 3-3

(A) Levels of inflammatory cytokines (IL-1β, IL-6, IL-10, TNF-α) in the colon and serum (n = 6).

(B) Presentation of mouse colons across different groups (n = 6).

(C) Bar chart depicting colon length (n = 6).

(D) Correlation analysis between inflammatory cytokines (IL-1β, IL-6, IL-10, TNF-α), serum markers associated with DLBCL disease burden (β2-MG, SF, and LDH), and intestinal microbial abundance.
(E) Correlation analysis between tryptophan metabolites and intestinal microbiota.
(F) Schematic representation of AB-PAS staining in the colon (Scale bars correspond to 50 μm (top) and 20 μm (bottom)).
(G) Cloud-rain plot depicting the relative abundance of *Parabacteroides*, *Ruminococcus*, and *Prevotella*.
(H) α-Diversity of microbial communities.
(I, J) Species composition at the phylum level and genus level.
Data are presented as mean ± SD. Statistical significance was determined by one-way ANOVA with Dunnett's multiple comparisons test or Mann-Whitney U test. ns, not significant, *$p < 0.05$, **$p < 0.01$, ***$p < 0.001$, ****$p < 0.0001$.

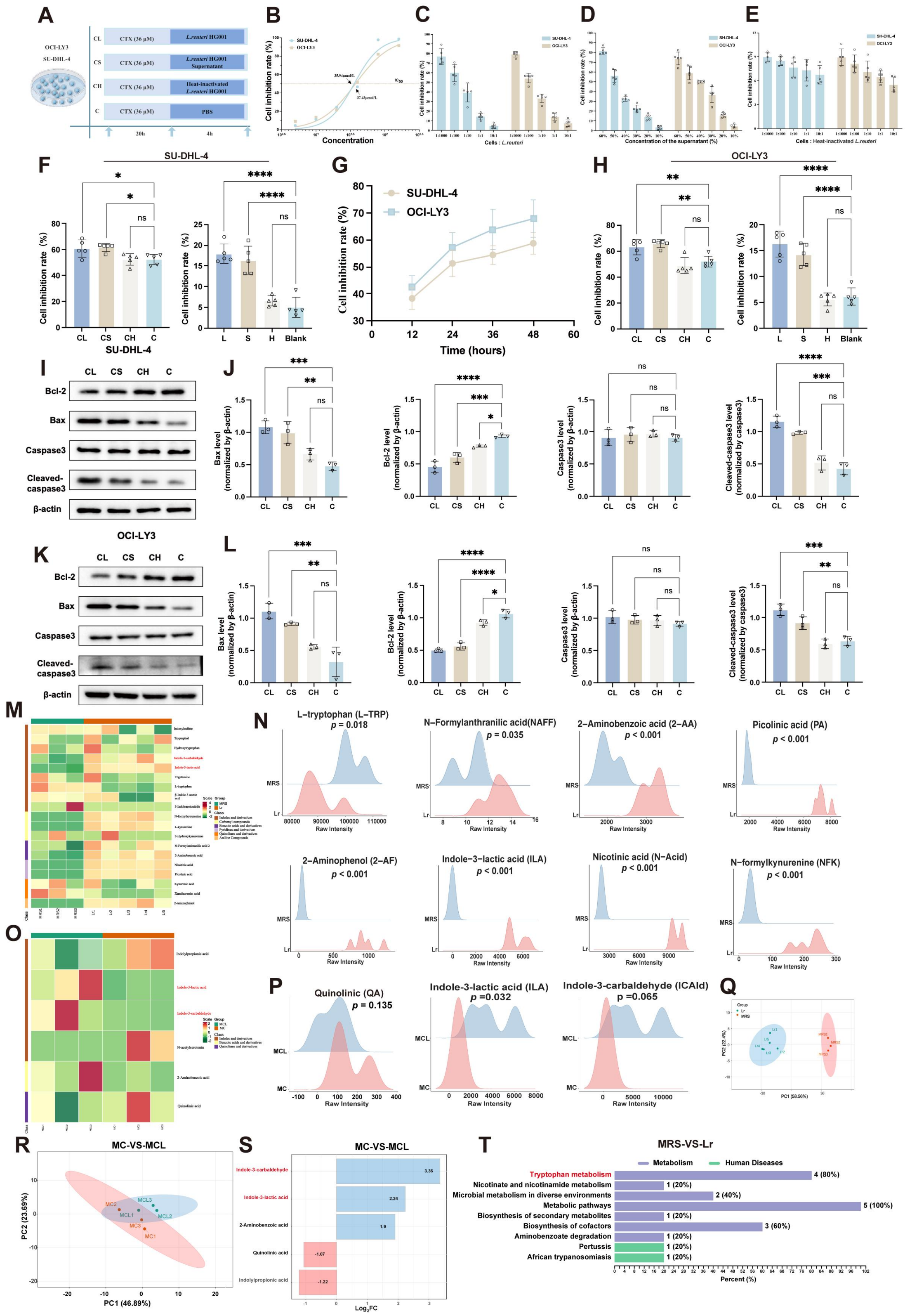

Supplementary Figure 4-1

The primary components of *L.reuteri* HG001. Synergizing with CTX for anti-DLBCL activity is present in the supernatant.

(A) Experimental design: Co-incubation of CTX (36 μM) with DLBCL cell lines (SU-DHL-4, OCI-LY3) for 20 h, followed by additional intervention with different components of *L.reuteri*

HG001. (live *L.reuteri* HG001, bacterial supernatant, heat-inactivated *L.reuteri* HG001.) for 4 h.
(B) IC_{50} curves of CTX-induced inhibition in DLBCL cell lines (SU-DHL-4, OCI-LY3).
(C-E) Inhibition profiles of DLBCL cell lines (SU-DHL-4, OCI-LY3) by varying concentrations of *L. reuteri*, supernatant of *L. reuteri*, and heat-inactivated *L.reuteri* HG001.
(F) Inhibition rates of SU-DHL-4 cells treated with CTX ± different components of *L.reuteri* HG001 (*L. reuteri*, supernatant of *L. reuteri*, and heat-inactivated *L.reuteri* HG001).
(G) Time-dependent inhibition curves of CTX on DLBCL cell lines (SU-DHL-4, OCI-LY3).
(H) Inhibition rates of OCI-LY3 cells treated with CTX ± different components of *L.reuteri* HG001.
(I-L) Representative Western blot images and quantitative bar charts evaluating apoptosis-related signaling molecules (Bax, Bcl-2, Cleaved-caspase3) in DLBCL cell lines (SU-DHL-4, OCI-LY3) post-intervention (n = 3).
(M) Heatmap of targeted tryptophan metabolomics analysis in *L.reuteri* HG001. supernatant.
(N) Ridge plots depicting relative concentrations of L-Tryptophan (L-TRP), N-Formylkynurenine (NAFF), 2-Aminoanthranilic acid (2-AA), Picolinic acid (PA), 2-Aminophenol (2-AF), Indole-3-lactic acid (ILA), Nicotinic acid (N-Acid), and N-Formylkynurenine (NFK) in bacterial supernatant.
(O) Heatmap of targeted tryptophan metabolomics analysis in mouse feces (MC group, MCL group).
(P) Ridge plots depicting relative concentrations of Quinolinic acid (QA), ILA, and ICAld in mouse feces.
(Q) PCA analysis of metabolic composition differences in bacterial supernatant.
(R) PCA analysis of metabolic composition differences in mouse feces (MC and MCL group).
(S) Bar charts illustrating metabolic composition differences in mouse feces (MC and MCL groups).
(T) KEGG enrichment analysis of metabolites in mouse feces (MC and MCL groups).
Statistical significance was determined using one-way ANOVA with Dunnett's multiple comparisons test and Mann-Whitney U test for intergroup comparisons. ns, not significant, $*p < 0.05$, $**p < 0.01$, $***p < 0.001$, $****p < 0.0001$.

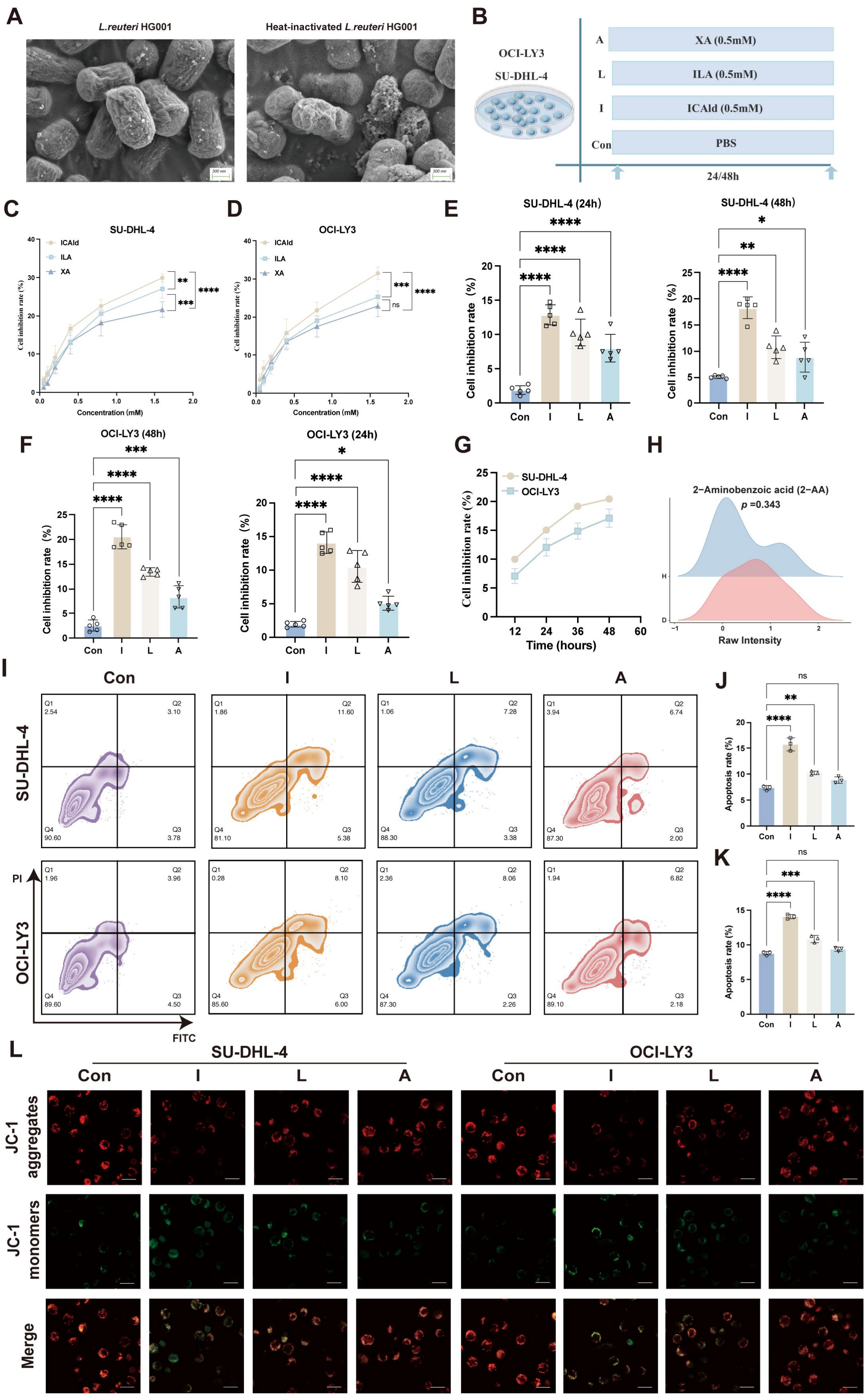

Supplementary Figure 4-2

(A) Electron microscopy images of *L.reuteri* HG001 and heat-inactivated *L.reuteri* HG001 (scale bar = 300 nm).
(B) Experimental design: Co-incubation of three primary beneficial metabolites of *L.reuteri* HG001. (ICAld, ILA, and XA, each at 0.5 mM concentration) with DLBCL cell lines (SU-DHL-4, OCI-LY3) for 24 h.
(C, D) Line graphs depicting inhibition rates of SU-DHL-4 and OCI-LY3 cells treated with varying concentrations of metabolites (ICAld, ILA, and XA).
(E, F) Bar charts illustrating inhibition rates of SU-DHL-4 and OCI-LY3 cells treated with metabolites (ICAld, ILA, and XA, 0.5 mM concentration) at 24 h and 48 h.
(G) Time-dependent bar charts (12 h, 24 h, 36 h, 48 h) showing inhibition rates of SU-DHL-4 and OCI-LY3 cells treated with metabolites (ICAld, ILA, and XA, 0.5 mM concentration).
(H) Ridge plot depicting relative serum concentrations of XA in humans.
(I-K) Flow cytometry analysis of apoptotic cells with a representative dot plot and quantified apoptotic proportion (n = 3).
(L) Schematic diagrams and quantitative bar charts (n = 3) of mitochondrial membrane potential alterations in DLBCL cell lines (SU-DHL-4, OCI-LY3) post-intervention with the three metabolites (ICAld, ILA, and XA) (scale bar = 20 μm).
Statistical significance was determined using one-way ANOVA with Dunnett's multiple comparisons test and Mann-Whitney U test for intergroup comparisons. ns, not significant, $*p < 0.05$, $**p < 0.01$, $***p < 0.001$, $****p < 0.0001$.

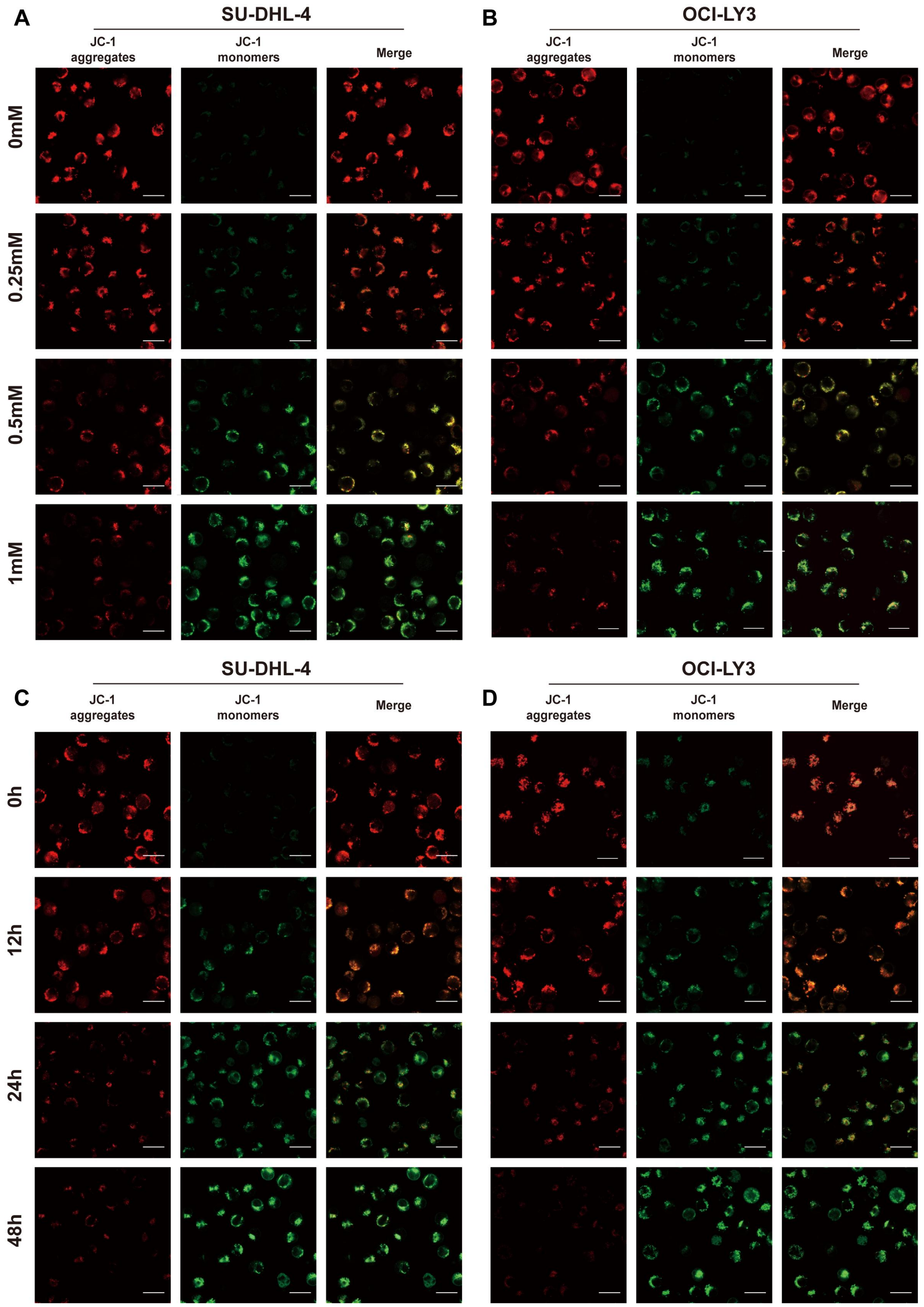

Supplementary Figure 4-3

(A, B) Schematic diagrams illustrating alterations in mitochondrial membrane potential of DLBCL cell lines (SU-DHL-4, OCI-LY3) following intervention with metabolic products (ICAld, ILA, XA) at varying concentration gradients (0 mM, 0.25 mM, 0.5 mM, 1.0 mM).

(C, D) Schematic diagrams depicting alterations in mitochondrial membrane potential of DLBCL cell lines (SU-DHL-4, OCI-LY3) following intervention with metabolic products (ICAld, ILA,

XA) at a concentration of 0.5 mM across different time gradients (0 h, 12 h, 24h, 48h) (scale bar = 20 μm).

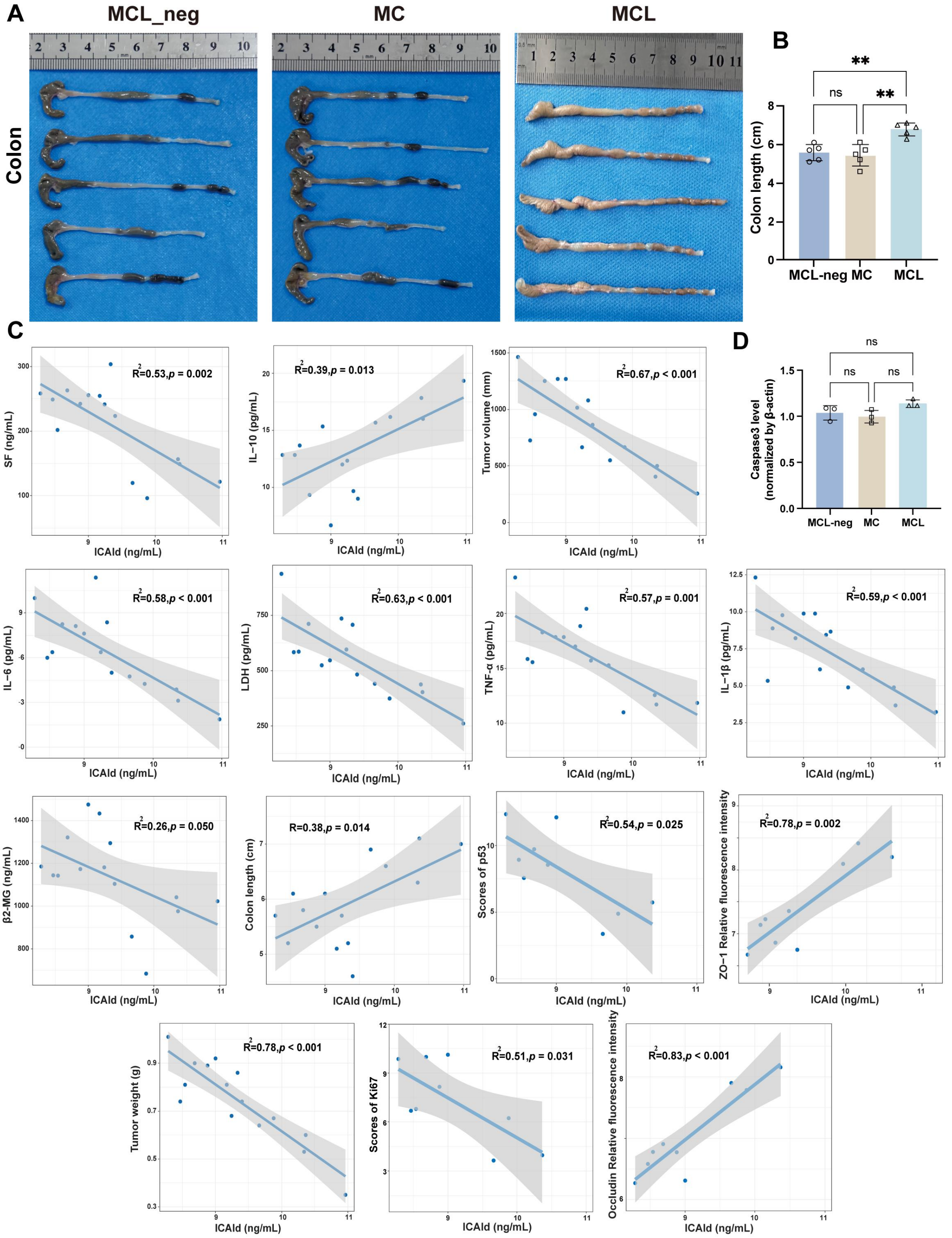

Supplementary Figure 5

(A) Representative images of mouse colons from each group at the conclusion of the intervention (n = 5).

(B) Bar charts depicting colon length (n = 5).

(C) Correlation analysis between serum ICAld levels and inflammatory cytokines (IL-1β, IL-6, IL-10, TNF-α), tumor progression-related hematological parameters (LDH, SF, β2-MG), colon length, tumor weight, tumor volume, proportions of Ki67- and P53-positive cells, and expression

levels of ZO-1 and Occludin in colonic tissues.

(D) Quantitative bar charts (n = 3) of Western blot analysis evaluating the expression of Caspase3. Statistical significance was determined using one-way ANOVA with Dunnett's multiple comparisons test and Mann-Whitney U test for intergroup comparisons. Pearson / Spearman linear correlation analysis was employed to examine the interrelationships among variables. ns, not significant, *$p < 0.05$, **$p < 0.01$, ***$p < 0.001$.

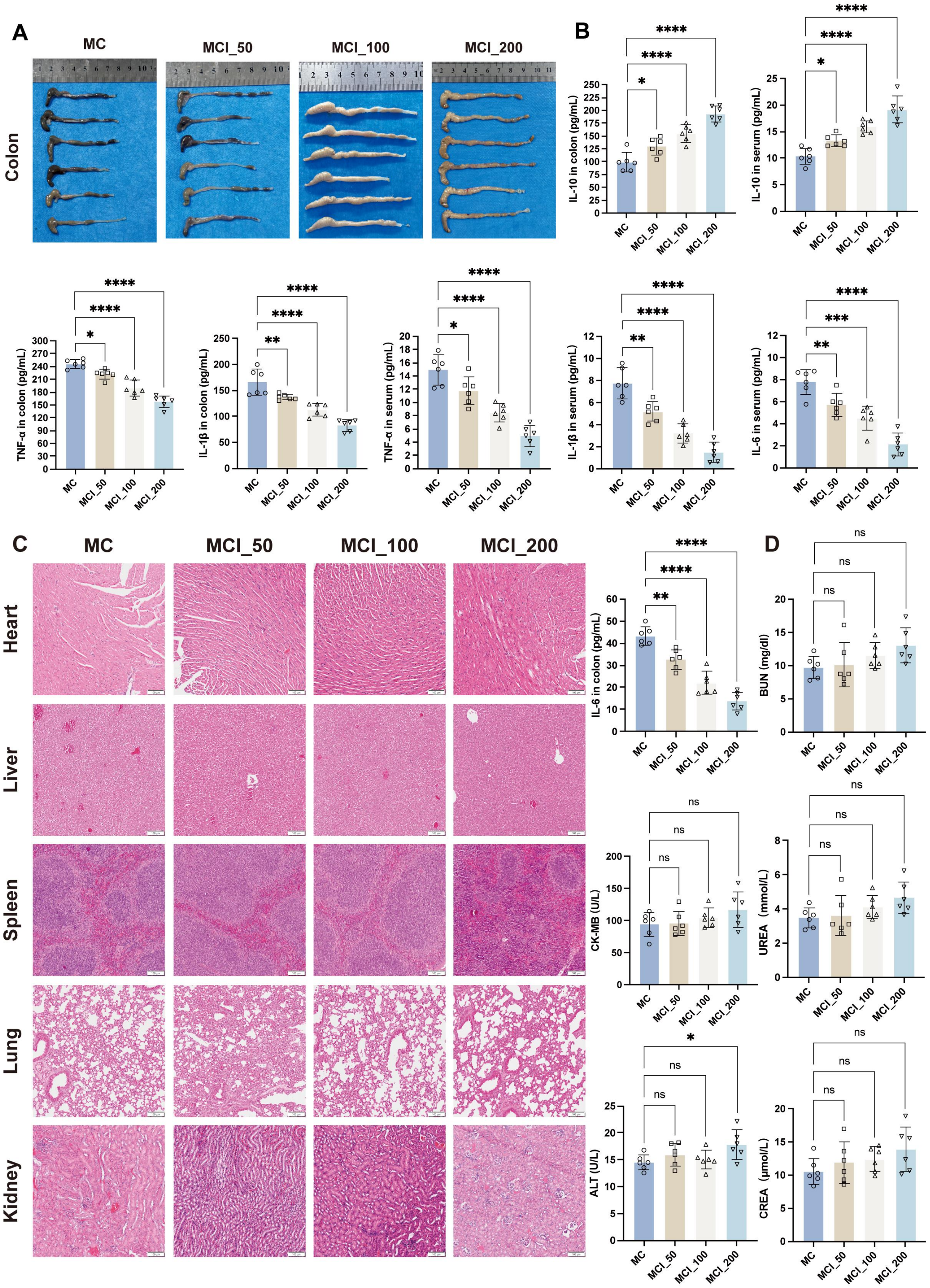

Supplementary Figure 6-1

(A) Representative images of mouse colons from each group at the conclusion of the intervention (n = 6).
(B) Levels of inflammatory cytokines (IL-1β, IL-6, IL-10, TNF-α) in serum and colon (n = 6).
(C) Schematic representation of HE staining for heart, liver, spleen, lung, and kidney tissues.
(D) Blood biochemical parameters (ALT, CK-MB, UREA, CREA, BUN; n = 6).
Data are presented as mean ± standard deviation. Statistical significance was determined using one-way ANOVA with Dunnett's multiple comparisons test and Mann-Whitney U test for intergroup comparisons. ns, not significant, $*p < 0.05$, $**p < 0.01$, $***p < 0.001$, $****p < 0.0001$.

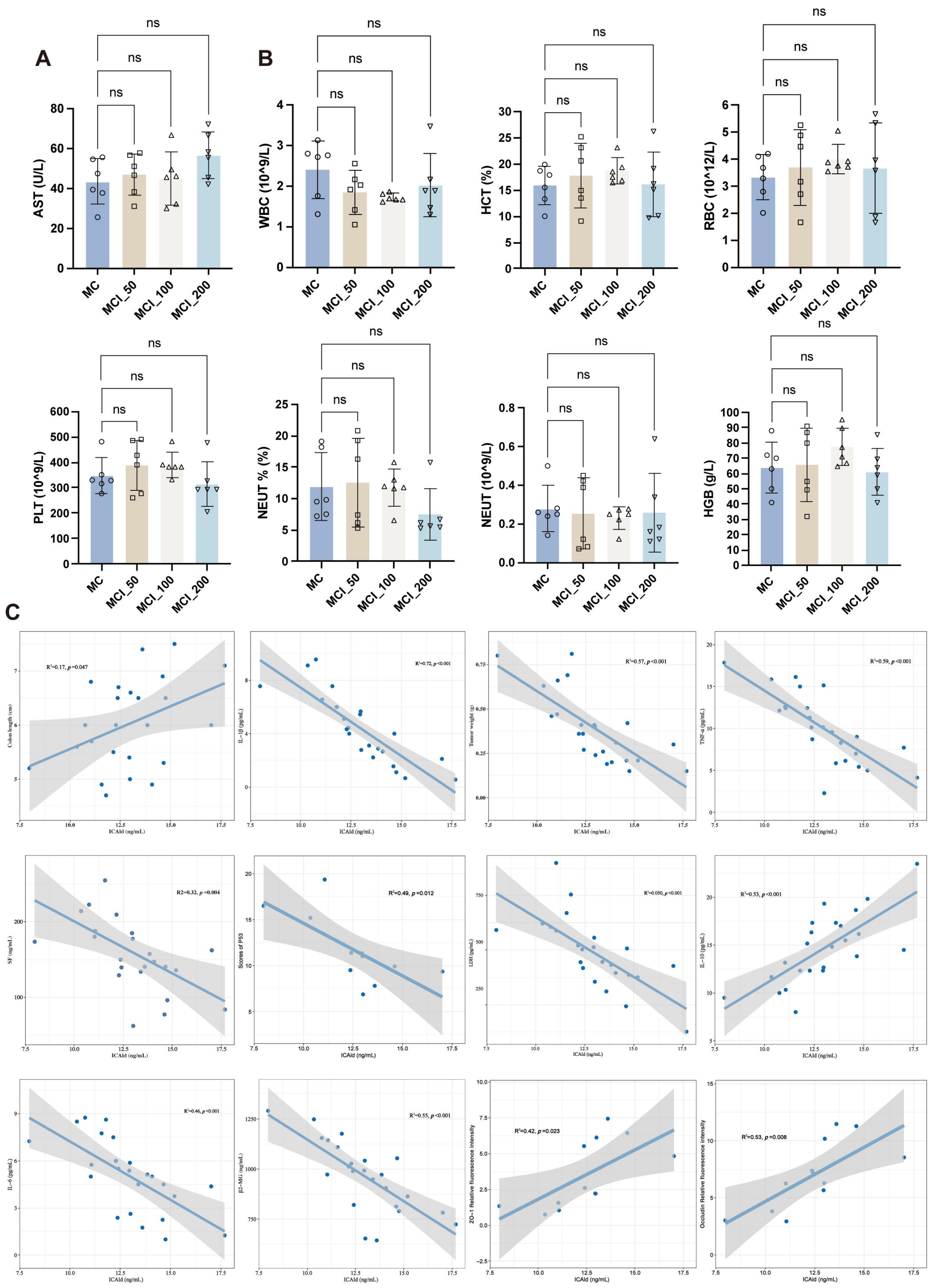

Supplementary Figure 6-2

(A) Blood biochemical parameters (AST, n = 6)

(B) Common hematological parameters (WBC, HCT, RBC, PLT, NEUT (%), NEUT, HGB, n = 6) in mice following the intervention.

(C) Correlation analysis between relative ICAld levels and intestinal parameters (ZO-1, Occludin, colon length), inflammatory burden (IL-1β, IL-6, IL-10, TNF-α), and tumor burden (tumor weight,

tumor volume, p53, Ki67, LDH, SF, β2-MG).

Statistical significance was determined using one-way ANOVA with Dunnett's multiple comparisons test and Mann-Whitney U test for intergroup comparisons, Pearson / Spearman linear correlation analysis was employed to examine the interrelationships among variables. ns, not significant, $*p < 0.05$, $**p < 0.01$, $***p < 0.001$, $****p < 0.0001$.

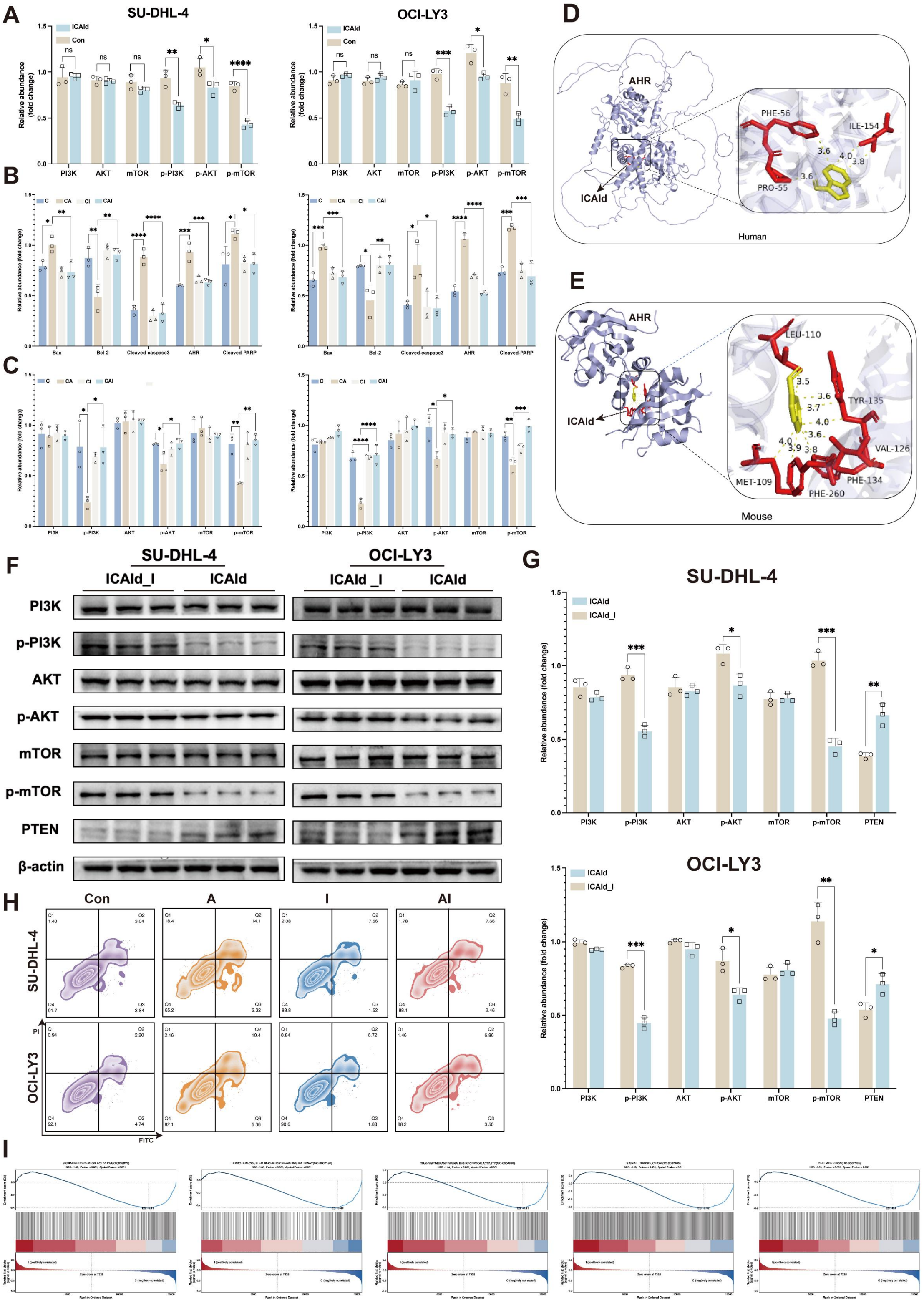

Supplementary Figure 7-1

(A) Quantitative bar charts (n = 3) of Western blot analysis evaluating key signaling molecules (p-PI3K, p-AKT, p-mTOR) of the PI3K-AKT-mTOR pathway in DLBCL cell lines (SU-DHL-4, OCI-LY3) post-ICAld intervention.

(B, C) Quantitative bar charts (n = 3) of Western blot analysis assessing key signaling molecules, including AHR, PI3K-AKT-mTOR pathway components (p-PI3K, p-AKT, p-mTOR), and apoptosis-related markers (Bax, Bcl-2, Cleaved-caspase3, p-PARP) in DLBCL cell lines (SU-DHL-4, OCI-LY3) post-intervention with ICAld ± CH-223191 + CTX.

(D, E) Molecular docking diagrams of the AHR protein with ICAld (murine and human).

(F, G) Representative Western blot images and quantitative bar charts (n = 3) evaluating key signaling molecules (PTEN, p-PI3K, p-AKT, p-mTOR) of the PI3K-AKT-mTOR pathway in DLBCL cells post-intervention with ICAld ± CH-223191 (ICAld_I and ICAld group)

(H) Representative flow cytometry plots depicting apoptosis ratios in DLBCL cells post-intervention with ICAld ± CH-223191.

(I) GSEA enrichment analysis plots.

Statistical significance was determined using one-way ANOVA with Dunnett's multiple comparisons test and Mann-Whitney U test for intergroup comparisons. ns, not significant, $*p < 0.05$, $**p < 0.01$, $***p < 0.001$, $****p < 0.0001$.

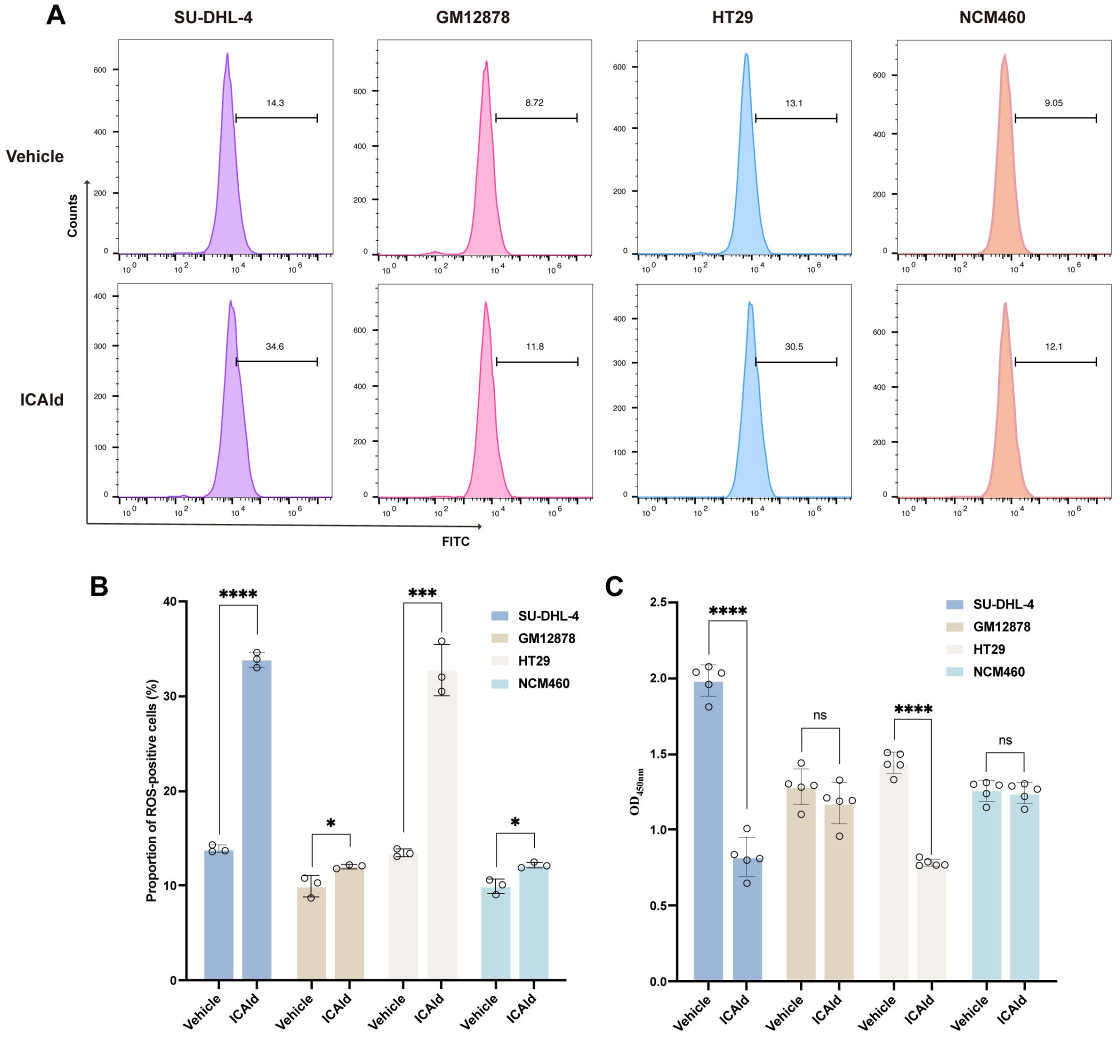

Supplementary Figure 7-2.

(A, B) Representative flow cytometry plots and quantitative analysis of the proportion of ROS-

positive cells in different cell lines (SU-DHL-4, GM12878, HT29, NCM460) following treatment with or without ICAld (0.5 mM) for 12 hours (n = 3).

(C) Cell proliferation in different cell lines (SU-DHL-4, GM12878, HT29, NCM460) following treatment with or without ICAld (0.5 mM) for 12 hours (n = 5). Statistical significance was determined using independent samples t-test or Mann-Whitney U test. ns, not significant, $*p < 0.05$, $***p < 0.001$, $****p < 0.0001$. (GM12878: Normal human B lymphocytes; HT29: Human colorectal adenocarcinoma cells; NCM460: Normal human colon epithelial cells).

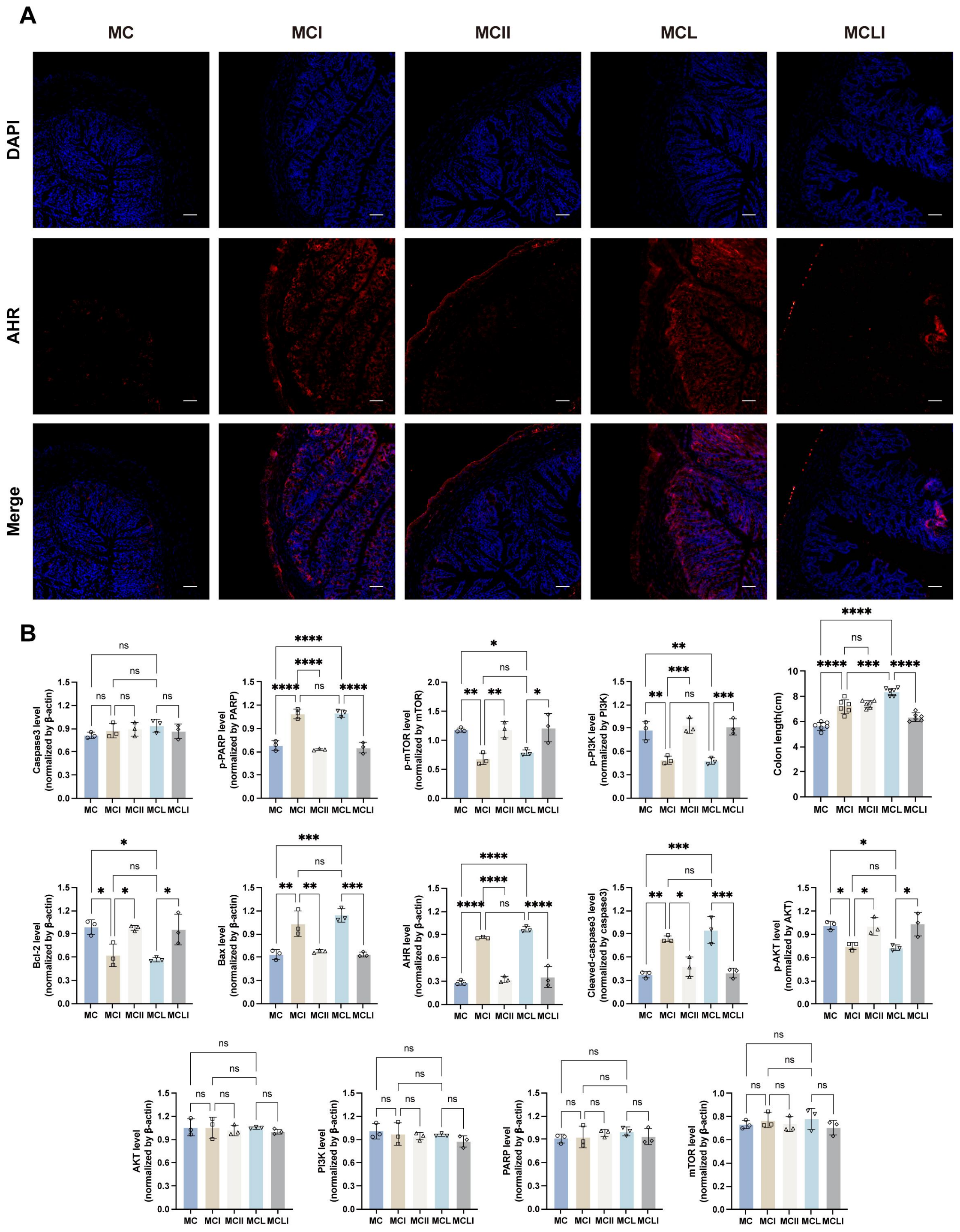

Supplementary Figure 8-1

(A) Schematic representation of AHR fluorescence staining in colonic tissues (scale bar = 50 μm).

(B) Quantitative bar charts (n = 3) of Western blot analysis evaluating the expression of AHR protein, PI3K/AKT/mTOR pathway components (p-PI3K, p-AKT, p-mTOR, PTEN), and apoptosis-related signaling molecules (Bax, Bcl-2, Cleaved-caspase3, p-PARP) in DLBCL tumor tissues.

Statistical significance was determined using one-way ANOVA with Dunnett's multiple comparisons test and Mann-Whitney U test for intergroup comparisons. ns, not significant, *$p < 0.05$, **$p < 0.01$, ***$p < 0.001$, ****$p < 0.0001$.

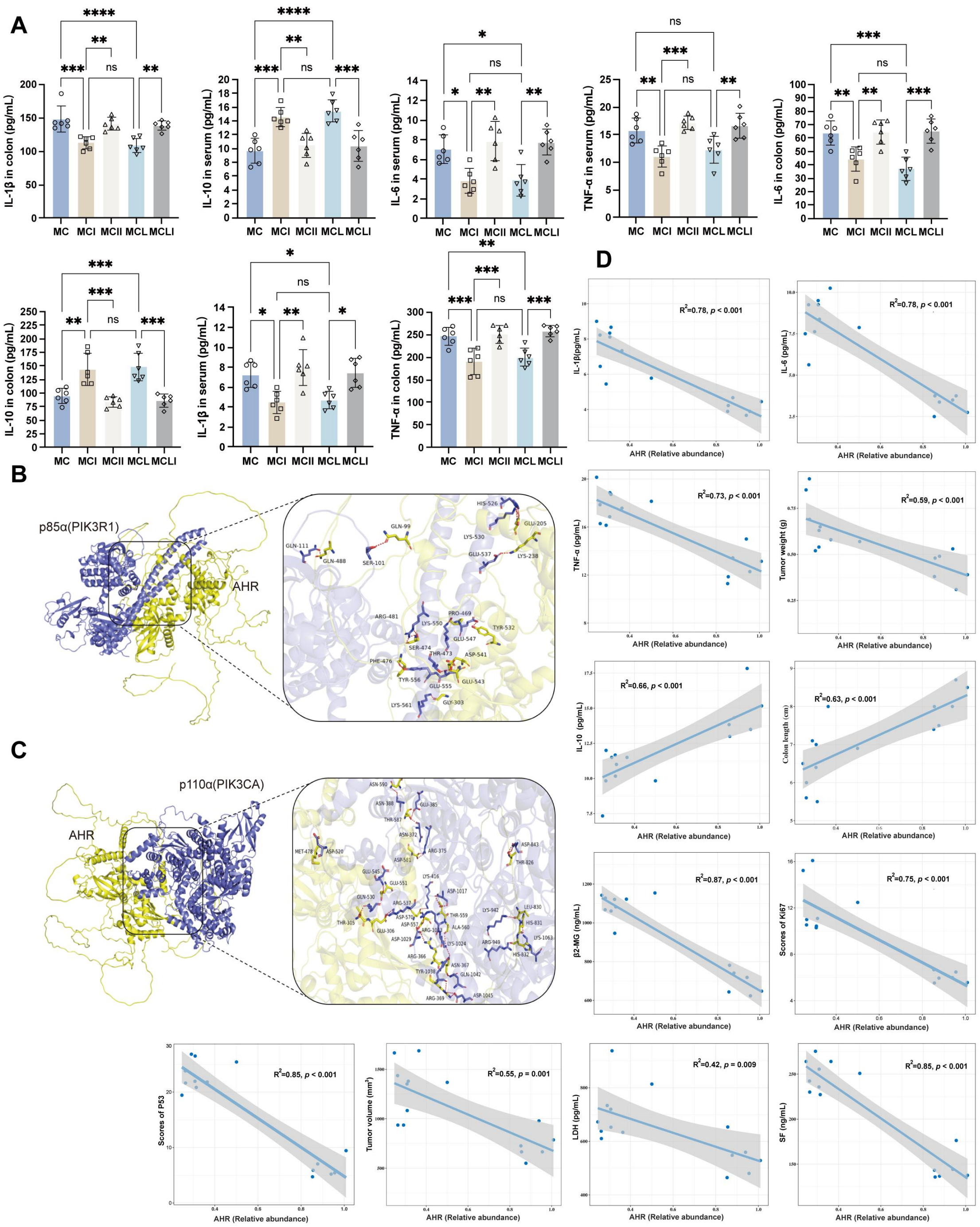

Supplementary Figure 8-2

(A) Levels of inflammatory cytokines (IL-1β, IL-6, IL-10, TNF-α) in serum and colon (n = 6).

(B) Molecular docking diagram of AHR with the PI3K (p85α subunit).

(C) Molecular docking diagram of AHR with the PI3K (p110α subunit).

(D) Correlation analysis between relative AHR content in DLBCL tumor tissues and indicators such as colon length, inflammatory burden (IL-1β, IL-6, IL-10, TNF-α), and tumor burden (tumor weight, tumor volume, p53, Ki67, LDH, SF, β2-MG).

Statistical significance was determined using one-way ANOVA with Dunnett's multiple comparisons test and Mann-Whitney U test for intergroup comparisons. Pearson / Spearman linear correlation analysis was employed to examine the interrelationships among variables. ns, not significant, *$p < 0.05$, **$p < 0.01$, ***$p < 0.001$, ****$p < 0.0001$.

Table S1 Baseline Clinical Characteristics of the Enrolled Patients (Group D) and Healthy Controls (Group H)

**Variables**	Total (n = 40)	Group D (n = 21)	Group H (n = 19)	*p* value
Age (years)	59.00 ± 11.01	58.81 ± 12.19	59.21 ± 9.87	0.910
BMI (kg/m²)	22.28 ± 3.02	22.27 ± 3.46	22.29 ± 2.54	0.981
BSA (m²)	1.56 ± 0.20	1.59 ± 0.21	1.52 ± 0.19	0.280
Pulse (bpm)	78.50 ± 11.68	78.14 ± 10.81	78.89 ± 12.87	0.842
Sex				
Male	27 (67.50)	14 (66.67)	13 (68.42)	0.906
Female	13 (32.50)	7 (33.33)	6 (31.58)	
Family history of cancer				
No	39 (97.50)	20 (95.24)	19 (100.00)	1.000
Yes	1 (2.50)	1 (4.76)	0 (0.00)	
Smoking history				
No	17 (42.50)	9 (42.86)	8 (42.11)	0.962
Yes	23 (57.50)	12 (57.14)	11 (57.89)	
Drinking history, n (%)				
No	25 (62.50)	13 (61.90)	12 (63.16)	0.935
Yes	15 (37.50)	8 (38.10)	7 (36.84)	
Hypertension				
No	24 (60.00)	12 (57.14)	12 (63.16)	0.698
Yes	16 (40.00)	9 (42.86)	7 (36.84)	
Diabetes				
No	26 (65.00)	13 (61.90)	13 (68.42)	0.666
Yes	14 (35.00)	8 (38.10)	6 (31.58)	

BMI: body mass index; BSA: Body Surface Area.

Measurement data are presented as the mean ± standard deviation (SD). Differences between groups were assessed using the unpaired t-test for normally distributed continuous variables, the Mann-Whitney U test for non-normally distributed variables, and the chi-square (χ^2) test for categorical variables. A two-sided *p*-value of < 0.05 was considered statistically significant for all tests.

Table S2 Baseline Clinical Characteristics of the Enrolled Patients (Group D)

Clinical characteristics		Cases
Sex		
	Male	14（66.7）
	Female	7（33.3）
Age (years)		
	<60	12（57.1）
	≥60	9（42.9）
Pathological subtype		
	GCB	12（57.1）
	Non-GCB	9（42.9）
Clinical stage		
	I	4（19）
	II	2（9.5）
	III	3（14.3）
	IV	12（57.1）
DEL		
	DELS	9（42.9）
	Non-DELS	6（28.6）
Ki67		
	≥50%	7（33.3）
	<50%	14（66.7）
Bcl-2		
	≥50%	12（57.1）
	<50%	9（42.9）
P53		
	≥50%	13（61.9）
	<50%	8（38.1）
Bcl-6		
	Positive	11（52.4）
	Negative	10（47.6）
CD79a		
	Positive	12（57.1）
	Negative	9（42.9）
CD10		
	Positive	12（57.1）
	Negative	9（42.9）
CD20		
	Positive	16（76.2）

	Negative	5（23.8）
C-MYC		
	Positive	13（61.9）
	Negative	8（38.1）
B2-MG (μg/mL)		
	≥2.7	3（14.3）
	< 2.7	18（85.7）
LDH (μ/L)		
	≥200	5（23.8）
	<200	16（76.2）
IPI score		
	≤1 point	5（23.8）
	2 points	3（14.3）
	3 points	5（23.8）
	≥4 points	8（38.1）
Efficacy assessment after four courses of treatment		
	CR	4（19）
	PR	1（4.8）
	SD	7（33.3）
	PD	9（42.9）
Risk Stratification		
	Low-risk	5（23.8）
	Intermediate-risk	8（38.1）
	High-risk	8（38.1）

GCB: Germinal Center B-cell-like DLBCL; DEL: Double-expression Lymphoma (Immunohistochemical detection: MYC≥40%, BCL2≥50%); C-MYC: Cellular myelocytomatosis oncogene; B2-MG: β2-microglobulin; LDH: Lactate Dehydrogenase; IPI: International Prognostic Index; CR: Complete Response.

Table S3 Antibodies, Reagents, and Drugs Used in the Study			
**Antibodies**	**Vendor**	**Catalog#**	**Concent ration**
PI3 Kinase p110 Alpha Monoclonal antibody	Proteintech	67071-1-Ig	1:1,000
AKT Antibody	Proteintech	10176-2-AP	1:5,000
AHR Monoclonal Antibody	Proteintech	67785-1-Ig	1:5,000
ZO-1 Polyclonal Antibody	Proteintech	21773-1-AP	1:500
Occludin Polyclonal Antibody	Proteintech	13409-1-AP	1:500
Ki67 Polyclonal Antibody	Proteintech	27309-1-AP	1:10,000
p53 Monoclonal Antibody	Proteintech	60283-2-Ig	1:500
β-Actin Monoclonal Antibody	Proteintech	66009-1-Ig	1:5,000
Bax Polyclonal Antibody	Proteintech	50599-2-Ig	1:5,000
HRP-conjugated Goat Anti-Mouse IgG(H+L)	Proteintech	SA00001-1	1:10,000
HRP-conjugated Goat Anti-Rabbit IgG(H+L)	Proteintech	SA00001-2	1:10,000
Phospho-PI3 Kinase p85 (Tyr458) Antibody	CST	17366S	1:1,000
Phospho-Akt (Ser473) Antibody	CST	4060S	1:2,000
Phospho-mTOR (Ser2448) Antibody	CST	5536S	1:1,000
PTEN (138G6) Antibody	CST	9559S	1:1,000
Caspase-3 Polyclonal Antibody	CST	9662S	1:1,000
Cleaved-Caspase 3 (Asp175), p17 Antibody	CST	9661S	1:1,000
PARP Antibody	Affinity	BF0719	1:1,000
Cleaved-PARP (Asp214) Antibody	Affinity	AF7023	1:1,000
Cleaved-Caspase 3 (Asp175), p17 Antibody	Abmart	TA7022	1:1,000
Bcl-2 Antibody	Abmart	T40056F	1:1,000
mTOR Antibody	abcam	ab32028	1:1,000
**Reagent kits**	**Vendor**	**Catalog#**	
Mitochondrial membrane potential detection kit (JC-1)	Beyotime	C2006	
Annexin V-FITC/PI Apoptosis Kit	Elabscience	E-CK-A211	
Mouse IL10 ELISA Kit ELISA Kit	BOSTER	EK0417	
Mouse IL-1 beta ELISA Kit	BOSTER	EK0394	
Mouse TNF Alpha ELISA Kit	BOSTER	EK0527	
Mouse IL6 ELISA Kit	BOSTER	EK0411	
Indole-3-carboxaldehyde ELISA Kit	MEIMAIN	MM-927392O1	
Mouse D-LDH (D-Lactate Dehydrogenase) ELISA Kit	Elabscience	E-EL-M0419	
Mouse SF ELISA Kit	ZCIBIO	ZC-38880	
Mouse-β2-microglobulin (β2-MG) ELISA Kit	Bioswamp	MU31656	
**Experimental drugs and reagents**	**Vendor**	**Catalog#**	
Indole-3-carboxaldehyde	aladdin	I1500225	
Indole-3-lactic Acid	aladdin	I157602	

Xanthurenic acid	MedChemExpress	HY-W014666
CH-223191	MedChemExpress	HY-12684
4-Hydroperoxy cyclophosphamide	MedChemExpress	HY-117433

Table S4 The Genomic Sequence of *Limosilactobacillus reuteri* HG001

**Strain**	**Sequence**
HG001	ACTTGGCAGGCGGGTGCTATACATGCAGTCGTACGCACTGGCCCAACTGAT<br>TGATGGTGCTTGCACCTGATTGACGATGGATCACCAGTGAGTGGCGGACG<br>GGTGAGTAACACGTAGGTAACCTGCCCCGGAGCGGGGGATAACATTTGGA<br>AACAGATGCTAATACCGCATAACAACAAAAGCCGCATGGCTTTTGTTTGAA<br>AGATGGCTTTGGCTATCACTCTGGGATGGACCTGCGGTGCATTAGCTAGTT<br>GGTAAGGTAACGGCTTACCAAGGCGATGATGCATAGCCGAGTTGAGAGAC<br>TGATCGGCCACAATGGAACTGAGACACGGTCCATACTCCTACGGGAGGCA<br>GCAGTAGGGAATCTTCCACAATGGGCGCAAGCCTGATGGAGCAACACCGC<br>GTGAGTGAAGAAGGGTTTCGGCTCGTAAAGCTCTGTTGTTGGAGAAGAAC<br>GTGCGTGAGAGTAACTGTTCACGCAGTGACGGTATCCAACCAGAAAGTCA<br>CGGCTAACTACGTGCCAGCAGCCGCGGTAATACGTAGGTGGCAAGCGTTA<br>TCCGGATTTATTGGGCGTAAAGCGAGCGCAGGCGGTTGCTTAGGTCTGATG<br>TGAAAGCCTTCGGCTTAACCGAAGAAGTGCATCGGAAACCGGGCGACTTG<br>AGTGCAGAAGAGGACAGTGGAACTCCATGTGTAGCGGTGGAATGCGTAGA<br>TATATGGAAGAACACCAGTGGCGAAGGCGGCTGTCTGGTCTGCAACTGAC<br>GCTGAGGCTCGAAAGCATGGGTAGCGAACAGGATTAGATACCCTGGTAGT<br>CCATGCCGTAAACGATGAGTGCTAGGTGTTGGAGGGTTTCCGCCCTTCAGT<br>GCCGGAGCTAACGCATTAAGCACTCCGCCTGGGGAGTACGACCGCAAGGT<br>TGAAACTCAAAGGAATTGACGGGGGCCCGCACAAGCGGTGGAGCATGTGG<br>TTTAATTCGAAGCTACGCGAAGAACCTTACCAGGTCTTGACATCTTGCGCT<br>AACCTTAGAGATAAGGCGTTCCCTTCGGGGACGCAATGACAGGTGGTGCA<br>TGGTCGTCGTCAGCTCGTGTCGTGAGATGTTGGGTTAAGTCCCGCAACGAG<br>CGCAACCCTTGTTACTAGTTGCCAGCATTGAGTTGGGCACTCTAGTGAGAC<br>TGCCGGTGACAAACCGGAGGAAGGTGGGGACGACGTCAGATCATCATGCC<br>CCTTATGACCTGGGCTACACACGTGCTACAATGGACGGTACAACGAGTCGC<br>AAACTCGCGAGAGTAAGCTAATCTCTTAAAGCCGTTCTCAGTTCGGACTGT<br>AGGCTGCAACTCGCCTACACGAAGTCGGAATCGCTAGTAATCGCGGATCA<br>GCATGCCGCGGTGAATACGTTCCCGGGCCTTGTACACACCGCCCGTCACAC<br>CATGGGAGTTTGTAACGCCCAAAGTCGGTGGCCTAACCTTTATGGAGGAGC<br>CGCCTAAGCGACGCGTTTCTCA

中国微生物菌种保藏管理委员会普通微生物中心
China General Microbiological Culture Collection Center(CGMCC)
地址： 北京市朝阳区北辰西路1号院3号 中国科学院微生物研究所 邮政编码：100101
电话：010-64807596 电子邮件：patent@im.ac.cn http://www.cgmcc.net

受理通知书（收据）
存活性报告书

用于专利程序的生物材料保存

受理日期 2025年10月20日

（请求保藏人或代理人的姓名、地址）

陈廷涛
南昌大学
江西省南昌市红谷滩区南昌大学前湖校区转化医学研究院

本保藏中心登记入册编号
CGMCC No. 36248

你（们）提供的请求保藏并注明以下鉴定参考的生物材料（株）：

Limosilactobacillus reuteri HG001

上述请求保藏的生物材料（株）附有
☐ 科学描述
√ 建议的分类命名

罗伊特氏粘液乳杆菌 ***Limosilactobacillus reuteri***

申请专利的发明名称

申请号NO.
申请日期 年 月 日

该生物材料（株）已于2025年10月20日由本保藏中心收到，并登记入册。
根据你（们）的请求，由该日起保存三十年，在期满前收到提供生物材料样品的请求后再延续保存五年。
该生物材料（株）的存活性经本保藏中心于2025年10月20日检测，结果 是：
（1）存活 √ （2）失活 /

中国微生物菌种保藏管理委员会普通微生物中心

签字(盖章)：喻亚静 日期：2025年10月31日
